# Exploring and mitigating potential bias when genetic instrumental variables are associated with multiple non-exposure traits in Mendelian randomization

**DOI:** 10.1007/s10654-022-00874-5

**Published:** 2022-05-27

**Authors:** Qian Yang, Eleanor Sanderson, Kate Tilling, Maria Carolina Borges, Deborah A. Lawlor

**Affiliations:** 1 MRC Integrative Epidemiology Unit at the University of Bristol, Bristol, UK; 2 Population Health Sciences, Bristol Medical School, University of Bristol, Bristol, UK; 3 National Institute for Health Research Bristol Biomedical Centre, University Hospitals Bristol NHS Foundation Trust and University of Bristol, Bristol, UK

**Keywords:** Mendelian randomization, Confounding, Selection bias, Pleiotropy, Causal diagram, UK Biobank

## Abstract

With the increasing size and number of genome-wide association studies, individual single nucleotide polymorphisms are increasingly found to associate with multiple traits. Many different mechanisms could result in proposed genetic IVs for an exposure of interest being associated with multiple non-exposure traits, some of which could bias MR results. We describe and illustrate, through causal diagrams, a range of scenarios that could result in proposed IVs being related to non-exposure traits in MR studies. These associations could occur due to five scenarios: (i) confounding, (ii) vertical pleiotropy, (iii) horizontal pleiotropy, (iv) reverse causation and (v) selection bias. For each of these scenarios we outline steps that could be taken to explore the underlying mechanism and mitigate any resulting bias in the MR estimation. We recommend MR studies explore possible IV—non-exposure associations across a wider range of traits than is usually the case. We highlight the pros and cons of relying on sensitivity analyses without considering particular pleiotropic paths versus systematically exploring and controlling for potential pleiotropic or other biasing paths via known traits. We apply our recommendations to an illustrative example of the effect of maternal insomnia on offspring birthweight in UK Biobank.

**Supplementary Information:**

The online version contains supplementary material available at 10.1007/s10654-022-00874-5.

## Introduction

Mendelian randomization (MR) is a special case of instrumental variable (IV) analysis where single nucleotide polymorphisms (SNPs) randomly allocated at conception are proposed as IVs [1]. MR requires three core assumptions to provide a valid test of the causal null hypothesis: first, IVs are strongly associated with an exposure of interest (relevance); second, there are no common causes between IVs and the outcome of interest (independence); and third, IVs influence the outcome only through the exposure (exclusion restriction) [1, 2]. While the relevance assumption can be tested, the independence and exclusion restriction assumptions are impossible to verify and only their plausibility can be explored [3]. Additionally, a fourth assumption is required to quantify the magnitude of the average causal effect of exposure on outcome using MR [2, 4, 5]. The fourth assumption has two versions that are frequently used: homogeneity (exposure-outcome effect does not depend on the proposed IV) and monotonicity (proposed IVs cannot increase exposure level in some participants while decrease it in others) [2, 4, 5].

With increasing sample sizes and more extensive coverage of the phenome in genome-wide association studies (GWAS), SNPs are increasingly found to associate with multiple traits [6–8]. Many different mechanisms could result in genetic IVs for an exposure of interest being associated with multiple non-exposure traits, some of which could bias MR results. Understanding whether the association of genetic IVs with non-exposure traits indicates a violation of core MR assumptions (i.e. independence and exclusion restriction) is challenging given the underlying causal structure for these associations is unknown. A large and increasing body of literature has focused on methods for exploring and dealing with unbalanced horizontal pleiotropy [9, 10], with these methods widely used in MR studies. Few MR studies have formally investigated potential mechanisms underlying the associations of proposed genetic IVs with non-exposure traits and explored whether such associations reflect mediation of the exposure-outcome effect, even though appropriate methods for MR mediation analysis are available [11, 12]. We aim to provide recommendations on how to leverage knowledge of existing associations between proposed genetic IVs and non-exposure traits to systematically evaluate the impact of various potential causal structures on bias in MR analysis, and to use this to inform appropriate sensitivity analyses to mitigate bias.

This paper is laid out as follows. In “Scenarios that could explain associations of genetic IVs with multiple traits”, we use directed acyclic graphs (DAGs) to illustrate five scenarios that could result in an association of a proposed genetic IV with a non-exposure trait and highlight when this could bias MR analysis. In “Recommendations for exploring scenarios that result in IV-non-exposure trait associations and obtaining unbiased MR estimates”, we describe different methods for discriminating between scenarios and methods for mitigating against potential bias for both one- and two-sample MR. In “Real data example”, we apply our recommendations to an MR analysis exploring the potential causal relationship between maternal insomnia and offspring birthweight in the UK Biobank (UKB). In “Discussion”, we end with a discussion.

## Scenarios that could explain associations of genetic IVs with multiple traits

**Fig. 1:**
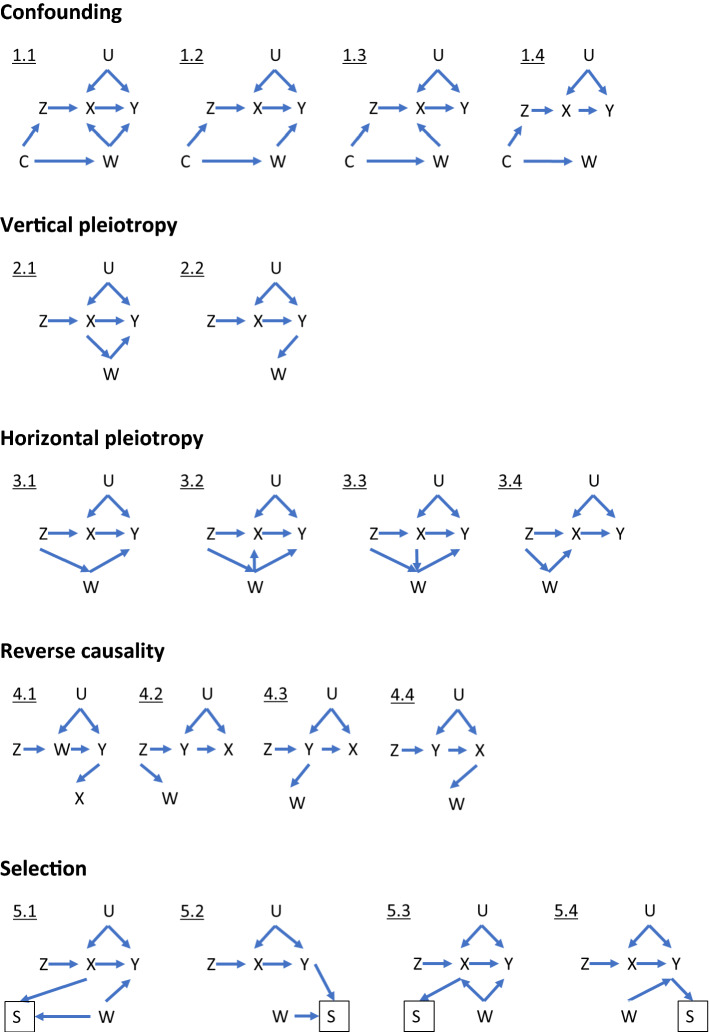
Directed acyclic graphs illustrating scenarios when an unexpected genetic instrumental variable-non-exposure trait association could be observed. Z: genetic instrumental variable; X: exposure of interest; Y: outcome of interest; U: unmeasured confounders; W: non-exposure traits; C: confounding factors, e.g. population stratification, cryptic relatedness and assortative mating; S, selection. For simplicity, we use single nodes even when there may be multiple variables, and these scenarios do not consider time-varying exposures and critical/sensitive-period exposure effects [2, 17]. Scenarios illustrated by 1.1, 1.2, 3.1–3.3, 5.1–5.4 would be expected to bias the MR estimate of X–Y effect; 1.3, 1.4, 2.1, 2.2 and 3.4 would not; 5.2 and 5.4 would be unbiased under the null

We describe five scenarios consistent with proposed genetic IVs being associated with non-exposure traits: (i) confounding (Fig. 1 (DAGs 1.1–1.4); (ii) vertical pleiotropy (DAGs 2.1 and 2.2), (iii) horizontal pleiotropy (DAGs 3.1–3.4), (iv) reverse causality (DAGs 4.1–4.4) and selection (DAGs 5.1–5.4). We discuss below how each of these scenarios could result in proposed genetic IVs (Z) being associated with non-exposure traits (W) and when this might bias MR estimates of the effect of the exposure of interest (X) on the outcome of interest (Y). In this study, we assume that X, W and the effects of Z on them do not vary with time, given MR examines genetic predisposition to X across a large part of the life course and might be unable to distinguish critical or sensitive-period exposure effects [13, 14]. If the effects of Z on X or W do vary over the lifecourse, MR results would be interpreted as the estimate of the causal effect of changing liability to X across the lifecourse (rather than the causal effect of X at the timepoint it is measured) on Y. The interpretation of those MR results is not the focus of this paper and has been explored elsewhere [15–17].

Phenotypic confounders cannot influence an individual’s germline genotype since genotypes are determined at conception and do not change throughout life. However, there are several phenomena that might confound genotype – phenotype associations at the population level by influencing the distribution of genotypes and phenotypes in a population. These would include as population structure, assortative mating, dynastic effects, and linkage disequilibrium (LD) [18–21]. *Confounding* could result in Z – W associations in the presence of a common factor that influences the distributions of Z and W (DAGs 1.1 to 1.4). Population structure can confound Z–W associations if there are systematic differences in the distributions of Z and W between subgroups of individuals in the same sample due to different ancestry backgrounds, geographical location, or cryptic relatedness (inclusion of relatives in the same sample) [18]. Assortative (non-random) mating can occur when people choose their partners based on particular characteristics (e.g. height or educational attainment) and can be based on a single phenotype (i.e. single-trait assortative mating) or two different phenotypes (i.e. cross-trait assortative mating) [19]. Despite offspring genotype inheritance being random in relation to parent’s genotype, assortative mating leads to systematic differences in allele frequencies and potentially introduces confounding in genotype-phenotype (e.g. Z–W) associations at the population level [18]. Dynastic effects occur when parental genotype influences offspring phenotype beyond genetic inheritance (i.e. indirectly through parental phenotype) and can introduce confounding of offspring genotype–phenotype associations [18]. Linkage disequilibrium (LD) can confound Z–W association if Z is correlated with other genetic variants within the same locus, which influence W via an independent biological pathway [22]. If any of these sources of Z–W confounding is present, we would expect bias in MR estimates where W causes Y via a path that does not contain X (DAGs 1.1 and 1.2), but not otherwise (DAGs 1.3 and 1.4).

Pleiotropy refers to the association of a SNP with multiple traits, and has two types: vertical (also known as spurious or false) and horizontal (also known as genuine or true) [23]. In the scenario of *vertical pleiotropy* (DAGs 2.1 and 2.2), despite pleiotropic associations of Z with X and W, the effect of Z on Y is fully mediated by X. Therefore, the exclusion restriction assumption is not violated and we would not expect the MR result to be biased [24]. In the scenario of *horizontal pleiotropy* (DAGs 3.1–3.4), Z is a cause of X and W via independent biological pathways. When both X and W affect Y independently (DAGs 3.1–3.3), the MR estimate is expected to be biased. Horizontal pleiotropy is typically considered one of the main threats to the validity of MR studies since pleiotropy is a ubiquitous biological phenomenon [7, 8]. However, there is no bias in the MR estimate when W does not affect Y independently of X (DAG 3.4).

In the scenario of *reverse causality* (DAGs 4.1–4.4), Z primarily causes the hypothesised outcome Y, which in turn affects the hypothesised exposure X. As such, selection of Z as an IV for X would give a biased MR result for the effect of X on Y [25]. With respect to the focus of this paper, this would only result in an association of Z with W if Z directly or indirectly influences W (DAGs 4.1–4.4). We have included this scenario for completeness. However, we do not consider exploration of Z–W associations to be a good way of identifying causal directions between X and Y. Bidirectional MR and the Steiger directionality test should be more suitable MR methods for exploring causal directions between any two traits [25]; these are described briefly in recommendation 4 of the next section.

*Selection* could result in Z–W associations if the selection mechanism leads to conditioning on a common effect of Z (or its descendant) and W. Selection into a study is likely to be directly influenced by certain phenotypic characteristics and, as a consequence, indirectly affected by genotypes influencing these characteristics [26]. Therefore, we consider four illustrative mechanisms where selection could induce an association between Z-W, all of which could introduce bias in MR of X on Y: selection on (1) X and W (DAG 5.1), (2) Y and W (DAG 5.2), (3) X alone (DAG 5.3) and (4) Y alone (DAG 5.4). To reduce the number of DAGs, we have focused on DAGs (5.1–5.4) where Z–W associations can only be generated due to selection, and the MR estimates of X on Y would be expected to be biased because of the alternative pathways from Z via W to Y independent of X. The selection of participants could occur due to several different reasons, such as a selected response to joining a study, loss to follow-up, missing data, survival bias, conditioning on heritable characteristics either in the MR analyses or in the GWAS where summary data are selected for two-sample MR [27], or in studies of disease progression if both Z and W affect disease incidence [28]. Such selection is not specific to Z–W associations. For example, previous gene-by-environment MR studies of smoking heaviness identified selection bias due to stratifying on smoking status [29, 30]. Those studies calculated associations of proposed genetic IV with smoking status and with observed confounders (of smoking status-outcome effect), or explored the magnitude of selection bias via simulation [29, 30]. Methods to mitigate selection bias in previous MR included inverse probability weighting [31], multivariable Mendelian randomization (MVMR) adjusting for major causes of survival [32], residual-adjusted model [33] and Bayesian cluster-based model [34] to distinguish effects on disease prognosis from conditioning on its incidence. Selection bias in MR has been comprehensively explored in other papers [27, 28, 31, 35, 36] and will not be the focus of our paper.

## Recommendations for exploring scenarios that result in IV-non-exposure trait associations and obtaining unbiased MR estimates

Having described some different scenarios that could result in proposed genetic IVs (Z) for exposure relating to non-exposure traits (W) we now provide a list of recommendations for identifying W, and exploring the mechanism underlying the Z–W association. We then investigate whether the Z–W association is expected to bias the MR estimates and, if bias is expected, how it might be mitigated (Table 1 summarises the methods we recommend for mitigating bias).

**Table 1:** Approaches to explore the plausibility of the scenario and methods that can produce unbiased test of the causal effect under the corresponding scenario

Scenarios	One-sample MR with individual data	Two-sample MR with summary data
Population stratification	Check Z—population structure (e.g. principal components, birthplace, home location or study centre)	Check how the GWAS of X and Y dealt with population structure
	Adjust for population structure in Z–W and compare the adjusted estimates with crude estimates	Use negative control outcomes^a^
Cryptic relatedness	Estimate genetic similarity	Check how GWAS of X and Y dealt with cryptic relatedness
	Remove one individual from each genetically related pair from analyses	
Assortative mating	Check associations of individuals’ Z (for X) with their partner’s W and Z (for W)	Rely on previous evidence
	Adjust for parental Z for X in two-stage least squares	
Linkage disequilibrium	Use ‘robust methods’ when multiple SNPs from different regions are proposed as IVs	Use ‘robust methods’ when multiple SNPs from different regions are proposed as IVs
	Apply colocalization methods (e.g. HEIDI [22]) if few SNPs located in a single gene are proposed as IVs^b^	Apply colocalization methods (e.g. HEIDI [22]) if few SNPs located in a single gene are proposed as IVs^b^
Vertical pleiotropy	Univariable MR for W–Y, bidirectional MR between X and W, tests for heterogeneity between multiple Z^c^, IV inequalities test (for categorical exposure only)^d^	Univariable MR for W–Y, bidirectional MR between X and W and Steiger directionality test, tests for heterogeneity between Z^c^, MR-Egger intercept
	Method: Two-stage least squares (for continuous outcomes)	Method: Inverse variance weighted
Horizontal pleiotropy	Univariable MR for W–Y, bidirectional MR between X and W, tests for heterogeneity between multiple Z^c^, IV inequalities test (for categorical exposure only)^d^	Univariable MR for W–Y, bidirectional MR between X and W and Steiger directionality test, tests for heterogeneity between Z^c^, MR-Egger intercept
	Methods: Multivariable MR, sisVIVE, MR-GENIUS, MR-MiSTERI	Methods: Multivariable MR, MR-Egger, weighted median, weighted mode, MR-PRESSO, MR-TRYX

*GWAS* genome-wide association studies, *MR* Mendelian randomization, *W* non-exposure traits, *X* exposure of interest, *Y* outcome of interest, *Z* genetic instrumental variables

^a^Negative control outcome is assumed to share the same underlying confounding structure as the outcome of interest, but not be influenced by the exposure of interest[37]

^b^HEIDI assumes that there is a single causal SNP within the locus, and each other SNP shows an effect due to LD with the causal SNP[22, 23]

^c^We assume that multiple genetic IVs are unrelated, and an observed heterogeneity is due to different mechanisms of multiple IV-exposure associations[38]

^d^IV inequalities test would be limited by low sensitivity with a small number of proposed IVs but low computational burden, or by high computational burden with a large number of proposed IVs but high sensitivity[39]

We focus most of our discussion on methods that are applicable to MR analyses where multiple independent SNPs from different genomic regions are proposed as IVs. The rationale for focusing on MR analyses using multiple IVs is the increasing number of independent SNPs strongly associated with putative exposures given large-scale GWAS [6], the increasing availability of robust MR methods and sensitivity analyses that require multiple IVs [9] and the potential that some proposed IVs will relate to several non-exposure traits [7, 8].

### Recommendation 1: Searching more thoroughly for Z–W associations

To date many MR studies have explored associations of Z with potential confounders of exposure (X)–outcome (Y) associations [40–42]. For example, in a recent MR study of the effect of maternal adiposity on infant birthweight the extent to which the proposed genetic IVs related to maternal education and smoking (plausible confounders of maternal adiposity and infant birthweight) was explored [41]. Bias component plots can be used to compare the IV-confounder associations versus exposure-confounder associations [43, 44]. Exploring associations of Z with any risk factors for Y is more appropriate than focusing solely on potential X–Y confounders since factors that influence the distribution of Z and Y can bias MR analyses regardless of whether they are a direct cause of X.

There are two broad approaches that could be used to identify W associated with Z in either one- or two-sample MR. One is to use prior/existing knowledge about the key causes of Y to select which traits to explore in further sensitivity analyses. It would be important to use expert knowledge to focus on those causes of Y that are not part of the hypothesized causal path from X to Y, and then examine whether Z relates to any of those non-exposure causes of Y. The second is to undertake a hypothesis-free comprehensive genotype-to-phenotype (also known as phenome-wide) scan approach [45] using automated tools (e.g. PhenoScanner [46], GWAS Catalog [47], MR-Base [48] and PHESANT [49]). Such an approach helps gain insight about all possible measured non-exposure traits associated with Z. In the situation where we are using MR PheWAS to test potential genetic instrument specificity to explore potential violation of MR assumptions, as here, we would suggest less stringent multiple testing *p*-value threshold than used in studies where the primary aim is to explore the potential effect of an exposure on multiple outcomes (e.g. [50, 51]). The aim in this situation would be to minimise bias by identifying as many possible horizontal pleiotropic paths as possible and hence we would recommend using a *p*-value threshold of 0.05 (the conventionally used threshold with no correction for multiple testing) when undertaking PheWAS to identify. The first (knowledge-based) approach has the advantage that it is focused, potentially selects the risk factors for Y that have the strongest effects, and is likely to require fewer variables to be taken forward for subsequent analyses (as described in recommendations below). However, subject matter knowledge may be incomplete or fallible and so some Z–W associations may be missed. The second (phenome-wide) approach has the advantage that it is less limited by expert knowledge. However, this approach is still dependent on the completeness and quality of the data available, and typically relies on an arbitrary choice of a *p*-value threshold, which is not straightforward given the need to account for the high multiple testing burden, whilst still aiming to identify all potential pleiotropic pathways.

Whether the knowledge-based or the phenome-wide approach is preferable will depend on the specific research question, available data, and extent of subject matter knowledge. A combination of both could be done by a priori selection of hypothesised causes of Y (that are not part of the X–Y causal path) and exploring Z–W associations with those without taking account of multiple testing, but rather focusing on point estimate magnitudes and precision to decide which need to be explored further to decide where the associations might bias the MR results. That could be supplemented by a phenome-wide association study in which multiple testing adjustment was applied.

### Recommendation 2: Assessing the impact of confounding

Family-based MR designs can be used to test and mitigate bias due to confounding from multiple sources (i.e. relatedness, population stratification, assortative mating and dynastic effects) as previously described in detail [18–21]. However, this requires large-scale data on siblings or trios (mother, father and child) that may not be available.

In the absence of large-scale family data, we recommend exploring associations of Z with as many indicators of population structure as possible, such as geographical (e.g. region, longitude/latitude of birth and residence, and study centre) and ancestry (e.g. self-reported ethnicity and genetic principal components of ancestral background) characteristics. If a Z–W association attenuates substantially after adjustment for geographical/ancestral characteristics, that would suggest that the Z–W association is primarily driven by confounding due to population structure. The possibility of bias due to cryptic relatedness should be minimized by the use of established methods for identifying relatedness [52] and including genetically unrelated individuals in the analysis.

In two-sample MR, there is widespread use of pre-generated summary association results from GWAS, which limits the assessment of potential bias due to population structure. Newly developed ‘robust’ MR methods are unlikely to overcome bias due to population structure if no attempts have been made to control for this in the original GWAS [53]. Therefore, MR analysts should check and report whether the original GWAS used appropriate methods to account for population structure and cryptic relatedness. The use of negative control outcomes in MR studies has been recently proposed to detect potential population stratification and could add to the sensitivity analyses exploring this type of bias [37].

In both one- and two-sample MR where family approaches are not possible to explore the presence of confounding due to assortative mating or dynastic effects, we would recommend that researchers try to identify external evidence of the magnitudes of spousal correlations for any observed W (and for X) even with non ‘visible’ traits such as biomarkers. These could provide some evidence of the likely magnitude and direction of bias. Evidence suggests that assortative mating is stronger for ‘visible’ traits, with reported spousal correlations within the first year of marriage that were low (0.03– 0.1) for physical measures (body mass index [BMI], blood pressure and heart rate) and higher (0.3–0.4) for education and health behaviours (smoking, alcohol consumption and exercise) [54]. Bias that could occur with familial related confounding (assortative mating or dynastic effects) is also illustrated in a recent MR study, which found that the standard population based MR analyses showing lower BMI and taller stature causing higher educational attainment were attenuated to the null in family MR, whereas the positive effects of BMI on type 2 diabetes and blood pressure were consistent in both population and family based MR designs [21].

Genetic colocalization methods have been developed to distinguish confounding by LD (distinct SNPs in the same genomic region, one affecting X and the other affecting W) from pleiotropy (a SNP shared between X and W) [10, 22, 55, 56]. Confounding by LD is more likely to be an important source of bias in MR analyses using a single or a few SNPs (as is commonly the case for molecular phenotypes—i.e. gene expression, epigenetic markers, protein, and metabolite concentrations) [57]—compared to MR analyses proposing multiple SNPs from different genomic regions as IVs. This is because confounding by LD is expected to affect specific variants in a sporadic way rather than all variants systematically [9] and, therefore, unbiased results could be obtained using the so-called robust MR methods as described in “Recommendation 6: Sensitivity analyses exploring and controlling for bias due to horizontal pleiotropy” below.

### Recommendation 3: Assessing bias due to horizontal pleiotropy by exploring the W–Y association

By definition, bias in MR analysis of X on Y due to horizontal pleiotropy via W requires that W influences Y and we recommend that MR (or suitable methods [13]) are used to explore this. MR can provide reliable evidence of the effect of W on Y if there are strong and valid genetic IVs for W (Z_W_), which may not be the case. In MR of W on Y (as in any MR), weak instrument bias would tend to bias the estimates towards the observational associations in one-sample MR and to the null in two-sample MR with non-overlapping samples and reduce statistical power and, consequently, the precision of the estimates [58]. If there is credible evidence from MR analysis that W does not affect Y, then one would be more confident that there should not be bias in MR estimates of the effect of X on Y due to horizontal pleiotropic pathways mediated by W. If there is evidence for W–Y effect, or it is not possible to determine this, then bidirectional MR of an effect of X–W versus W–X could be valuable (see next recommendation).

### Recommendation 4: Orienting causal directions of effect between X and W

Bidirectional MR can be conducted to explore causal directions between X and W (i.e. whether X causes W, or vice versa) in either one- or two-sample MR [59, 60]. If bidirectional MR provides no evidence for a causal effect of X on W or vice versa, this suggests that Z affects X and W via independent pathways and bias MR analyses when the effect of W on Y is independent of X (DAG 3.1). If bidirectional MR suggests that X causes W, W is likely to be a consequence of X and should not bias MR estimates of X on Y (DAGs 2.1 and 2.2). However, it is possible that W mediates the effect from Z to Y partly independently of X (DAG 3.3). By contrast, if bidirectional MR suggests that W causes X, the presence of bias in the MR analyses would depend on whether X completely mediates any effect of W on Y (DAG 3.4) or not (DAG 3.2).

Bidirectional MR is still a valid test of causal directions in the presence of bidirectional effects between X and W with additional assumptions that accommodate time-varying relationships between X and W [17, 61]. However, whilst we recommend this to orient the causal directions of effect between X and W, it can be misleading when there are marked difference in the magnitudes and statistical power between MR of X on W and MR of W on X. For example, if the magnitude or power for MR of W on X is low (relative to the magnitude or power for MR of X on W) it may appear that there is no causal effect of W on X even in the presence of such an effect. Those magnitudes may vary during lifecourse, and thus bidirectional MR may identify X causes W at some points and W causes X at others, but could not distinguish which comes first. Additionally, overlapping SNPs in the GWAS of X and W can make it unclear which SNPs to select as valid IVs for X and W in bidirectional MR [60]. In two-sample MR, Steiger directionality test might help to identify (independent) valid IVs for X or W by comparing the variance explained by each SNP in X to that in W—under the assumption that each trait is measured with the same error a valid IV for X should explain more variance in X than W (and vice versa) [62].

### Recommendation 5: Adjusting for potential horizontal pleiotropic effects via W

Where there is evidence (from recommendations 3 and 4 above) that there may be bias in the MR estimate of the effect of X on Y due to horizontal pleiotropy mediated by W (DAGs 3.1–3.3), MVMR can be used to test the presence of effect X on Y adjusting for W in one- and two-sample MR [63]. MVMR requires information on not only Z–X and Z_W_–W associations but also Z–W and Z_W_–X associations, which means two-sample MR studies using summary statistics require access to full summary statistics of the original GWAS. If W mediates the X–Y association (DAG 3.3), controlling for W in MVMR obtains the direct effect rather than the total effect of X on Y, while its total effect could be estimated by using a subset of SNPs only related to X [63]. If W interacts with X, MVMR can be extended to model such interaction in one-sample but not two-sample MR [64]. MVMR can be used to estimate direct effects of correlated traits on an outcome as long as the proposed genetic IVs independently strongly predict each trait. Limitations of MVMR, such as problems with weak instruments, have been extensively discussed in the literature (e.g. [16, 61, 63]). Although MVMR has been extended to apply to a time-varying X, the model to minimise potential horizontal pleiotropy by further including time-varying W is still under development [16].

### Recommendation 6: Sensitivity analyses exploring and controlling for bias due to horizontal pleiotropy

The methods described in this section can also be applied in tandem with MVMR, or when additional assumptions of MVMR may not hold. These sensitivity analyses have been discussed extensively in the literature (e.g. [2, 3, 9, 10]). We recommend initially assessing between SNP heterogeneity [10] even if SNPs are being combined into a single polygenic risk score (PRS). In one-sample MR heterogeneity is commonly explored by ‘overidentifying’ tests [38], while in two-sample MR (including MVMR) the Cochran’s Q statistic is an equivalent test [10, 65]. If X causes Y, and Z are valid IVs, we expect the Z–Y effect to be proportional to the Z–X effect across multiple SNPs. Therefore, heterogeneous individual SNP causal estimates are indicative of invalid IVs (even though they could also result from model misspecification). IV inequalities test can be used specially for categorical exposures and outcomes in one-sample MR, to falsify both the independence and exclusion restriction assumptions [2, 66]. However, this test cannot distinguish horizontal pleiotropy from confounding. There are an increasing number of MR methods developed for correcting for horizontal pleiotropy and a full description of all of these is beyond the scope of this paper. Table 2 summarises some commonly used methods (i.e. sisVIVE [67], MR-GENIUS [68] and MR-MiSTERI [69] for one-sample MR and MR-Egger [70], weighted median [71], weighted mode [72], MR-PRESSO [73], MR-TRYX [74] for two-sample MR). It is important to recognise that (i) heterogeneity tests and most of the sensitivity tests in Table 2 can only be used where there are multiple SNPs, (ii) some methods are statistically inefficient and (iii) most methods have been initially developed for two-sample MR and can be applied to large one-sample data (e.g. UKB), with fewer specific to one-sample MR. We would also recommend checking for outlier SNPs (i.e. SNPs that contribute disproportionately more than expected to the heterogeneity across individual SNP causal estimates) since these can bias estimates from some MR methods, particularly regression-based methods such as inverse variance weighted (IVW) and MR-Egger. Some MR methods have been developed to deal with outlying SNPs, which might be done by removing identified outliers (e.g. sisVIVE [67], MR-PRESSO [73]), downweighing their contribution (e.g. weighted-median [71] and weighted mode [72]) or adjusting for detected pleiotropic pathways from outlying SNPs (MR-TRYX [74])*.* Whilst these methods are useful, we would recommend using them in tandem with other main and sensitivity analyses (e.g. MVMR) and with attempts to understand as much about the biology of the outlying SNPs as possible. Without this understanding it is challenging to understand whether the outlying SNP is introducing horizontal pleiotropy (and might be best removed or down weighted) or is the most biologically reliable SNP and, therefore, the most credible instrument in the analysis [10].

**Table 2:** Summary of select sensitivity analyses for exploring bias due to horizontal pleiotropy in Mendelian randomization (MR)

Name	Brief description	MR assumptions	Other issues
*For one-sample MR*			
sisVIVE [67]	It is an extension to two-stage least squares, which incorporates LASSO penalization	(1) relevance; (2) independence; (3) the alternative to exclusion restriction: at least 50% of proposed IVs are valid; (4) monotonicity^a^	It works for continuous outcomes only, is computationally intensive, and the current implementation do not provide 95% CIs
MR-GENIUS [68]	It is a version of G-estimation which is robust to time-varying SNP-exposure associations, unmeasured confounding and violation of IV assumptions	(1) the alternative to relevance: proposed genetic IVs should strongly affect the variance rather than the mean of the exposure; both (2) independence and (3) exclusion restriction can be relaxed; (4) the alternative to homogeneity^b^: no additive interaction with unmeasured selection	Estimates on binary exposures have ambiguous units. Proposed genetic IVs often explain a small variance of the exposure
MR-MiSTERI [69]	It is another version of G-estimation for estimating the causal effect among compliers^a^, which is robust to time-varying SNP-exposure associations, unmeasured confounding and violation of IV assumptions	(1) relevance, (2) independence, and (3) exclusion restriction all can be relaxed; (4) the alternative to monotonicity^a^: exposure-outcome effect does not vary with proposed invalid IV on additive scale; selection bias due to confounding does not vary with proposed invalid IV on multiplicative scale; residual variance for outcome is heteroscedastic and thus varies with proposed invalid IV	Its R package can only be used for continuous exposure and outcome at present
*For two-sample MR*			
MR-Egger [70]	It allows a non-zero intercept to test unbalanced horizontal pleiotropy	(1) relevance; (2) independence; (3) the alternative to exclusion restriction: InSIDE^c^; (4) homogeneity^b^	It is sensible to outliers and tends to suffer from low statistical power
Weighted median [71]	It is defined as the median of a weighted empirical density function of the Wald ratio estimates	(1) relevance; (2) independence; (3) the alternative to exclusion restriction: at least 50% of weight comes from valid IVs; (4) homogeneity^b^	Nil
Weighted mode [72]	It calculates the weighted mode of the Wald ratio estimates	(1) relevance; (2) independence; (3) the alternative to exclusion restriction: zero modal pleiotropy assumption ^d^; (4) homogeneity^a^ or monotonicity ^b^	Researchers need to choose a bandwidth to obtain the clustering effect, and different bandwidths might provide inconsistent estimates [10]
MR-PRESSO [73]	It assesses horizontal pleiotropy based on the contribution of each SNP to heterogeneity and provides adjusted MR estimates by removing outlier SNPs	(1) relevance; (2) independence; (3) the alternative to exclusion restriction: InSIDE^c^ and outliers (identified via MR-PRESSO global test) are due to potential horizontal pleiotropy; (4) homogeneity^a^ or monotonicity^b^	After removing outlier SNPs, the standard errors would decrease. Therefore, it would be more likely to reject the null
MR-TRYX [74]	It assesses horizontal pleiotropy based on the contribution of each SNP to heterogeneity and attempts to adjust for their horizontal pleiotropic effects using extra publicly available GWAS from MR-Base	(1) relevance; (2) independence; (3) the alternative to exclusion restriction: outliers (identified via RadialMR [75]) are due to potential horizontal pleiotropy; (4) homogeneity^a^ or monotonicity^b^	GWAS from MR-Base may not cover the whole genome or conducted in the target population (e.g. only female participants)

*ACE* average causal effect, *CI* confidence interval, *GWAS* genome-wide association studies, *InSIDE* instrument strength independent of direct effect, *IV* instrumental variable, *SNPs* single nucleotide polymorphisms

^a^Monotonicity means proposed IV cannot increase exposure level in some participants while decrease it in others. Thus, MR quantifies the magnitude of ACE among the unknown subpopulation of compliers who are monotonically affected by the proposed IV (i.e. local ACE)[2, 4]

^b^Homogeneity means the exposure-outcome effect is homogeneous across population and does not depend on proposed IV. Thus, MR quantifies the magnitude of ACE for the reference population (i.e. global ACE)[2, 4]

^c^The strength of genetic IV – exposure association should not be correlated with the strength of the pleiotropic effects across proposed IVs[70]

^d^The majority of SNPs could be invalid providing that the set of SNPs which form the largest homogeneous cluster are valid[72]

## Real data example

We used MR to explore the effect of maternal insomnia on offspring birthweight as a motivating example (DAG shown in Supplementary Fig. 1). It has been suggested that having insomnia and other forms of sleep disturbance may be associated with lower offspring birthweight though results are inconsistent [76, 77]. We explored this question using data from women who participated in the UKB study [78]. UKB is a cohort of 503 325 men and women who were on the National Health Service registry, aged between 40 and 69 years and living within 25 miles from one of 22 assessment centres [78]. Supplementary Table 1 summarises how each variable used here was measured in UKB and coded in our example.

### Using (one- and two-sample) MR to explore the effect of maternal insomnia on offspring birthweight

For both one- and two-sample MR, we proposed 80 genome-wide significant SNPs (listed in Supplementary Data 1) from the largest GWAS of insomnia in women [79] as our genetic IVs. In one-sample MR, SNPs were combined into a PRS to explore the effect of maternal insomnia on offspring birthweight among genetically unrelated UKB women of European descent who reported frequency of insomnia, had experienced at least one live birth and reported the birthweight of their first live born child (N = 165 184). In two-sample MR, we randomly split those genetically unrelated women of European descent into two groups (Supplementary Fig. 2) to obtain SNP-specific summary statistics. We obtained both SNP–insomnia and SNP–birthweight results from both of the random sub-samples and then pooled results from analyses in which sample 1 was used for SNP–insomnia and sample 2 for SNP–birthweight with those in which sample 1 was used for SNP–birthweight and sample 2 for SNP–insomnia to avoid sample overlap [59]. Full details are available in Supplementary Methods.

In one-sample MR using two-stage least squares, having insomnia causes 87 g lower birthweight in offspring, but its confidence interval (CI: − 182, 7) slightly overlaps with the null. In two-sample MR, IVW suggested having insomnia is related to lower offspring birthweight ( − 124 g when comparing having versus not having insomnia, 95% CI: − 230, − 19). Our unweighted PRS for insomnia was very weakly associated with a higher odds of having live born babies (OR 1.0029 per one more allele, 95% CI: 1.0008, 1.0051, *P*-value = 0.007), having insomnia was also weakly associated with a higher probability of having live born babies (8.6 × 10^–2^, 95% CI: 2.3 × 10^–2^, 1.5 × 10^–1^, *P*-value = 0.007).

### Searching more thoroughly for Z–W associations (Recommendation 1)

**Fig. 2:**
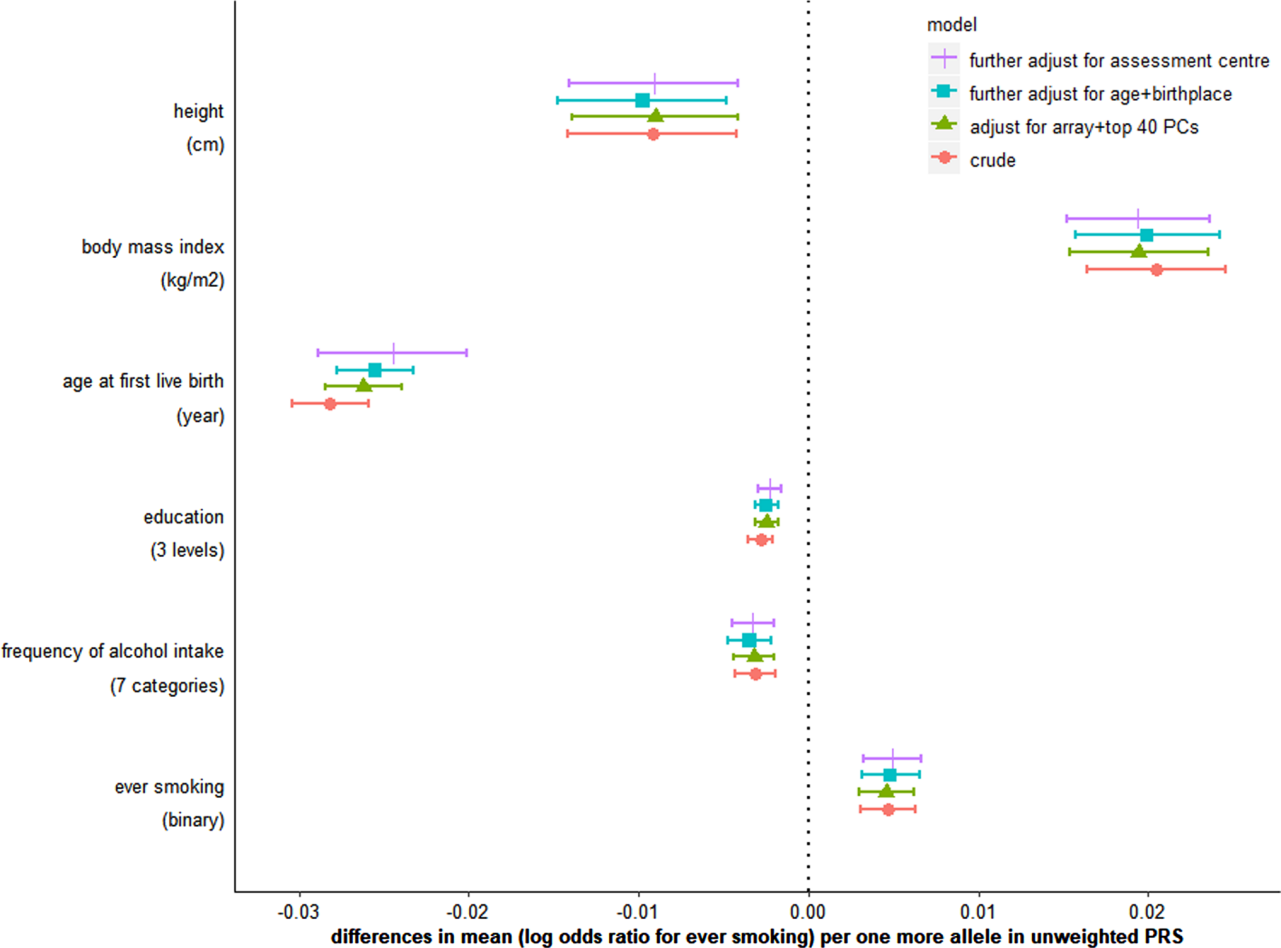
Associations of unweighted polygenetic risk score (PRS) for insomnia with six non-exposure traits before and after adjustment for population stratification. Supplementary Table 1 summarizes how education, frequency of alcohol intake and ever smoking are coded in this study

In this motivating example we only explored the six traits (i.e. maternal height, BMI, age at first live birth, education, frequency of alcohol intake and ever smoking) that we had a priori selected for checking, based on prior knowledge that these were key risk factors for variation in birthweight. We tested whether our PRS for maternal insomnia was associated with any of these traits and found that it was associated with all of them (Fig. 2). However, as noted in recommendation 1 above, there may be value in exploring a wider range of potential pleiotropic paths. Therefore, we undertook a comprehensive search for previously identified associations of the 80 SNPs in the insomnia PRS using Phenoscanner [46]. Supplementary Data 2 shows the results for 17,503 associations with 2844 traits meeting this threshold (*P*-value < 0.05). Associations of one or more of the insomnia SNPs were seen for insomnia, birthweight and all of our six a priori selected W (height, BMI, age at first live birth, education, frequency of alcohol intake, ever smoking). Other associations included traits related to our exposure or one of the a priori selected W. For example, several SNPs were associated with other sleep traits in addition to insomnia, and many were associated with a large number of different measures of adiposity that are available in Phenoscanner (e.g. total fat mass and fat mass of each body component, impedance, waist circumference). Additional non-exposure traits identified included mental health outcomes (e.g. depression, anxiety, mood swings, schizophrenia), musculoskeletal outcomes (bone mineral density, report of osteo or rheumatoid arthritis), respiratory/allergic outcomes (wheeze, asthma, eczema, lung function), reproductive outcomes (age at menarche) and general health (reported health rating, number of non-cancer diseases and number of prescribed medications). Some evidence of a statistical association was found for several related phenotypes showing they were unlikely to be due to chance. In the interest of space and clarity for this illustrative example we undertook further analyses on the risk factors for birthweight that we a prior listed above and that we see the proposed genetic IVs for insomnia are related to. In a focused applied paper these additional analyses would be done on all of the variables identified by the Phenoscanner.

### Exploring the role of confounding (Recommendation 2)

Each additional allele in the unweighted insomnia PRS was associated with a difference of −  0.004 (95% confidence interval [CI]: − 0.007, − 0.001) year in age at recruitment, − 3.7 × 10^–7^ (95% CI:  − 6.7 × 10^–7^, − 6.4 × 10^–8^) metre in longitude of birthplace and 2.1 × 10^–7^ (95% CI: 5.7 × 10^–8^, 3.5 × 10^–7^) metre in latitude of birthplace. There was evidence of some variation in the mean PRS across 22 UKB assessment centres (Supplementary Fig. 3; *P*-value = 9.2 × 10^–8^). After adjusting for genetic array, top 40 genetic principal components, participants’ age, birthplace and assessment centre, associations of the PRS with height, BMI, education, frequency of alcohol consumption and ever smoking were not attenuated, and its association with age at first live birth was slightly attenuated to the null (Fig. 2). In further MR analyses of age at first live birth, we obtained similar estimates before and after adjustment for participants’ age, birthplace and assessment centre (Supplementary Table 2). In the absence of spousal data, we were not able to directly assess assortative mating. From previous literature [19, 54] we would anticipate that assortative mating could occur for traits such as education, height, and lifestyle factors (e.g. smoking and alcohol), which might bias estimates for the effect of maternal insomnia on offspring birthweight if the assorted trait is genetically correlated with both the exposure and outcome of interest. Confounding by LD is unlikely to be a major source of bias in this example as our PRS consists of 80 SNPs from different genomic regions.

### Association of non-exposure traits with birthweight, testing causal direction between exposure and non-exposure traits and accounting for horizontal pleiotropy (Recommendations 3 to 5)

**Fig. 3:**
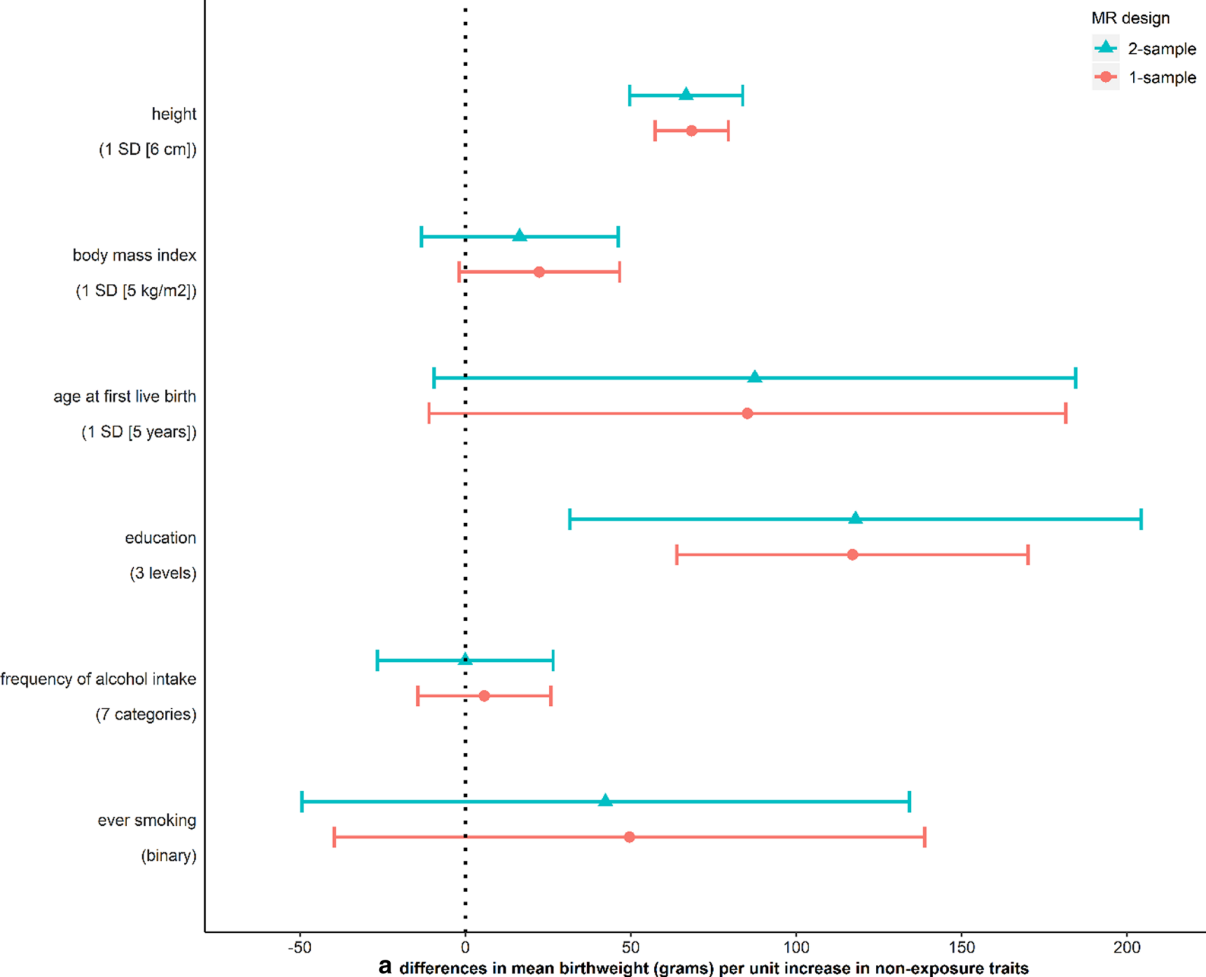

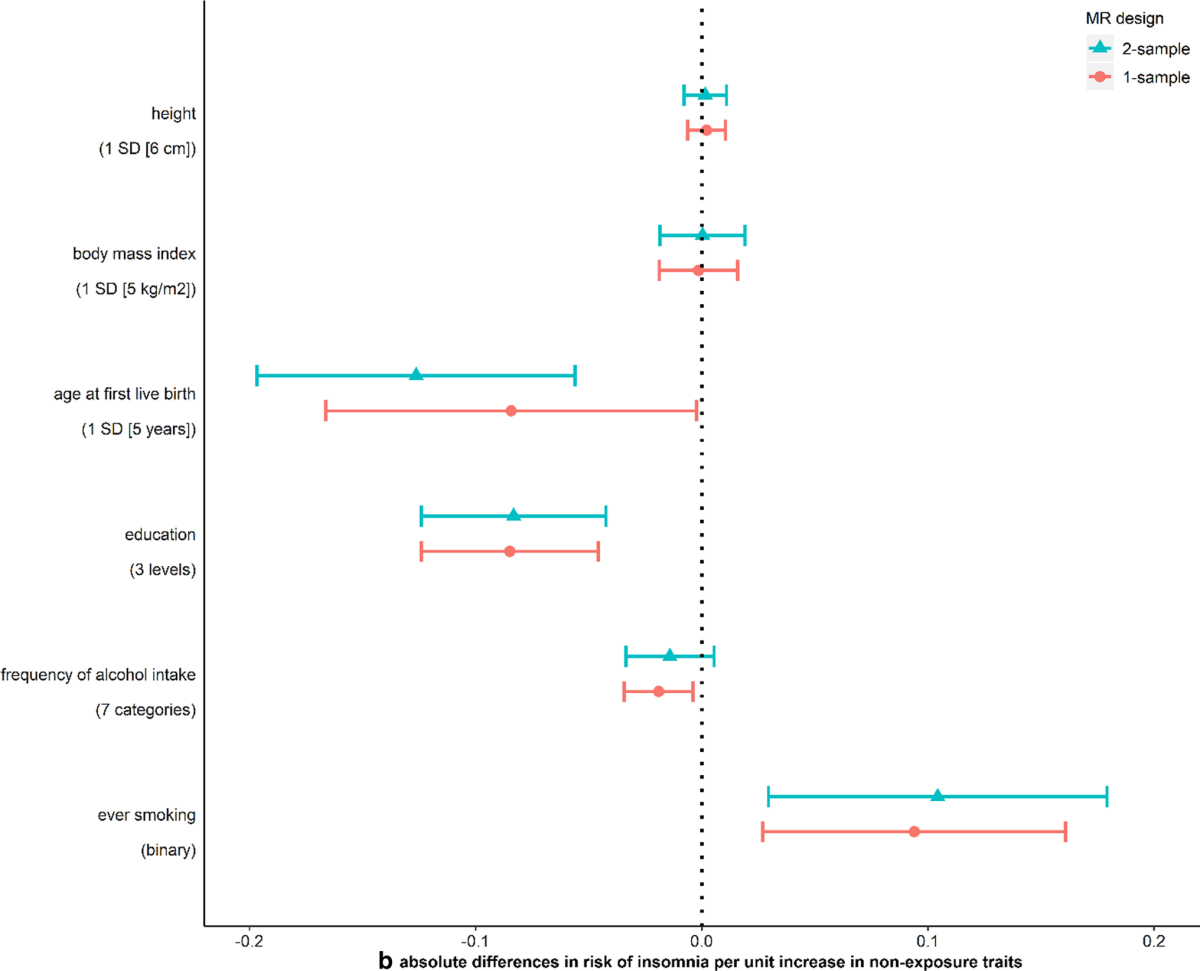

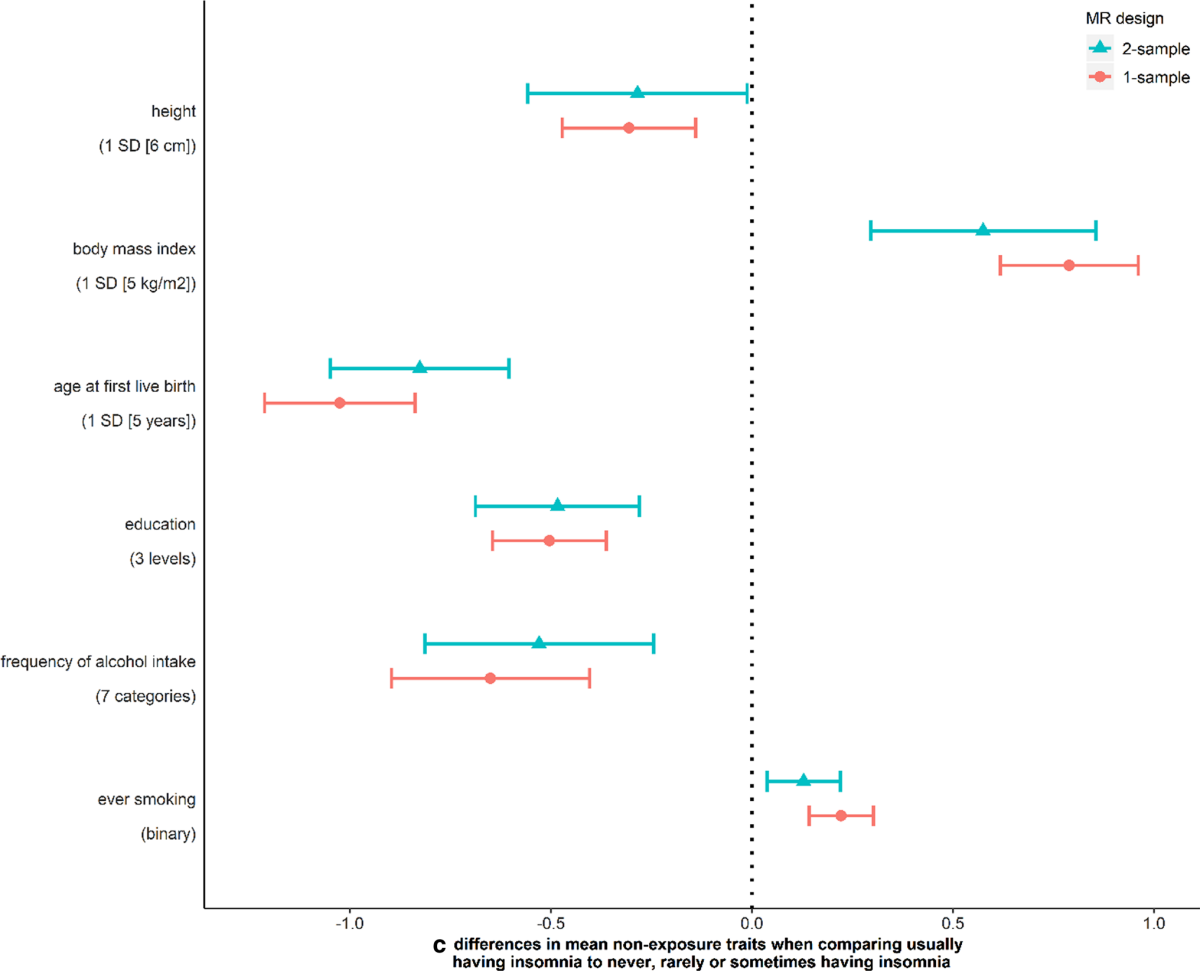
Mendelian randomization estimates for **a** non-exposure traits-birthweight (W–Y) effects, **b** non-exposure traits-insomnia (W–X) effects, and **c** insomnia-non-exposure traits (X–W) effects. “Usually” having insomnia is coded as 1, while “sometimes/rarely/never” having insomnia is coded as 0 (Supplementary Table 1)

**Fig. 4:**
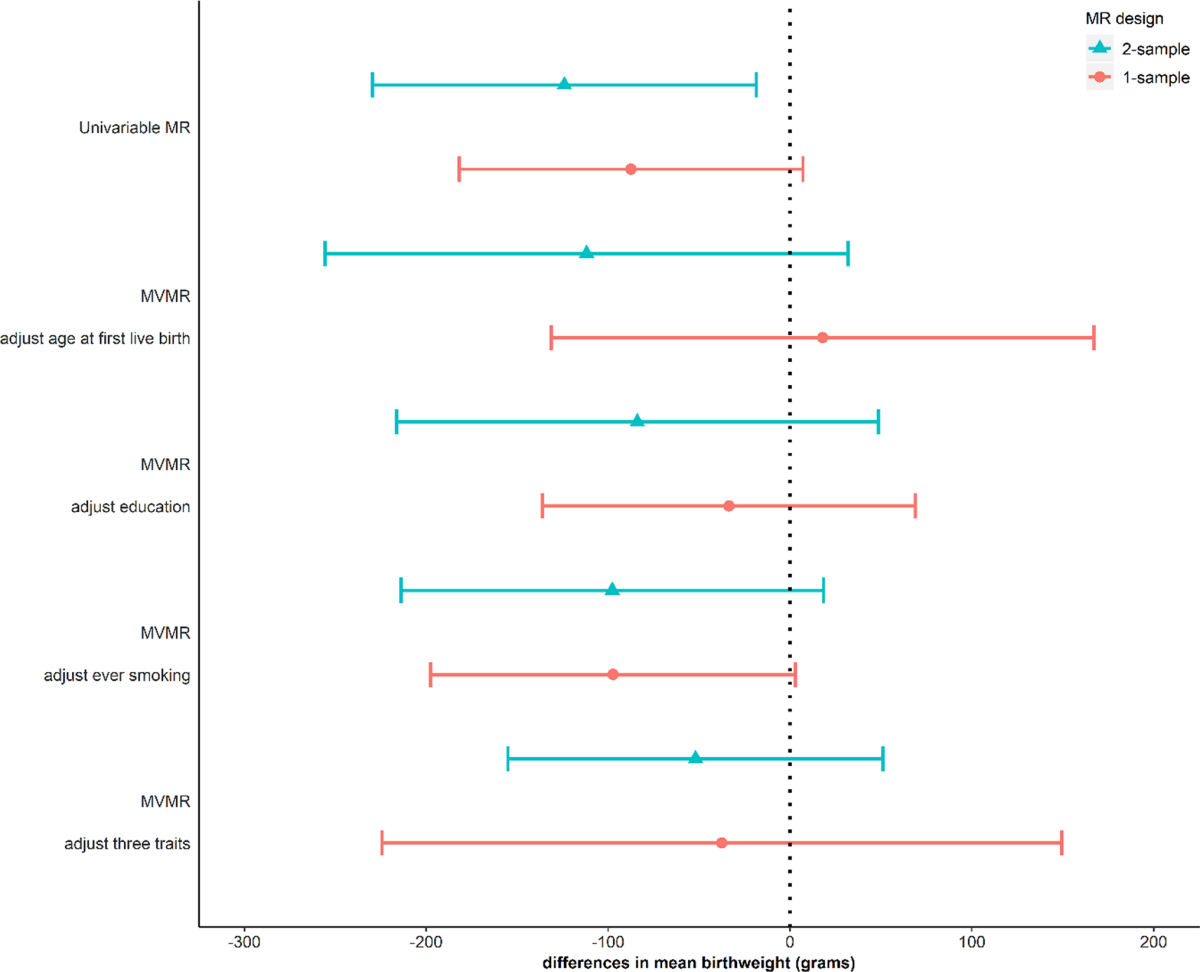
Multivariable Mendelian randomization (MVMR) estimates for the effect of maternal insomnia on offspring birthweight. Estimates are differences in mean birthweight when comparing reporting usually experiencing insomnia to never, rarely or sometimes experiencing it with and without adjustment for potential horizontal pleiotropy via maternal age at first birth, education and ever smoking

We use MR analysis to test the causal effect of each non-exposure trait on offspring birthweight. For these analyses, we identified proposed genetic IVs from published GWAS of height [80], BMI [81], age at first live birth [82] and education [83] in women independent of UKB and from previous GWAS of frequency of alcohol intake and ever smoking in UKB men and women [84]. Full details of the selected SNPs are provided in Supplementary Data 1. Under the MR assumptions, we found suggestive evidence of causal effects of five out of six non-exposure traits on birthweight despite some wide CIs (Fig. 3a). Height and BMI did not appear to cause insomnia, but for the other four traits MR showed evidence of causality in both directions, i.e. the traits caused, and were caused by insomnia (Fig. 3b and c). Because age at first live birth, education and ever smoking showed evidence of affecting both insomnia and birthweight, they were included in MVMR to estimate the direct effect of insomnia on birthweight adjusting for these three variables. After adjusting for them, the effect estimates of insomnia on birthweight attenuated towards the null compared to univariable MR (Fig. 4), though results are imprecise.

### Sensitivity analyses to explore unbalance horizontal pleiotropy (Recommendation 6)

**Fig. 5:**
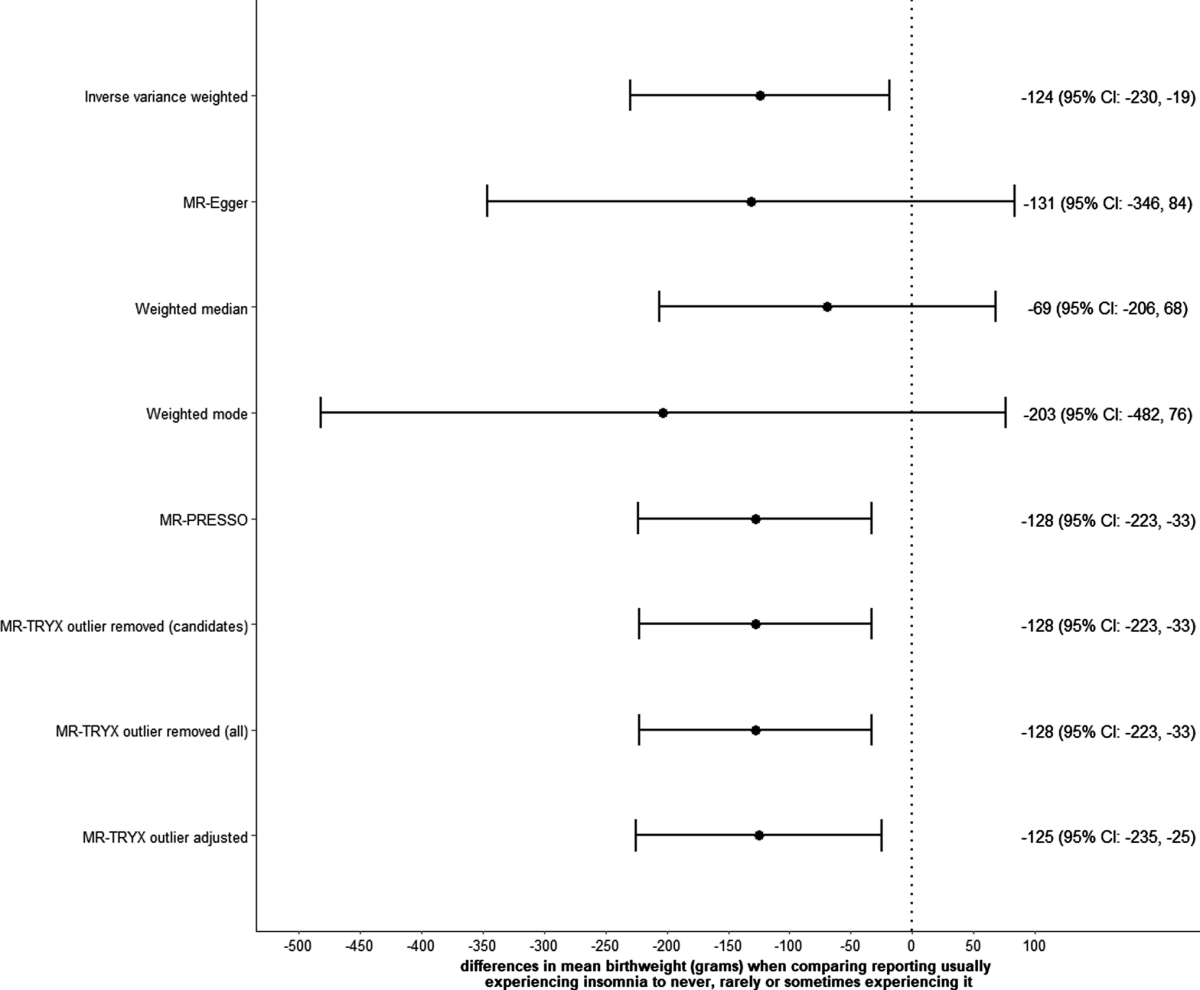
Sensitivity analyses for the effect of maternal insomnia on offspring birthweight using two-sample Mendelian randomization (MR)

In one-sample MR, Sargan test suggests invalidity among the 80 SNPs (Supplementary Table 3). Our sisVIVE (full results in Supplementary Data 3) showed that the association of insomnia with birthweight was greater than seen with univariable two-stage least squares, but MR-GENIUS provided a smaller estimate ( − 28 [95% CI: − 155, 100] g). In the two-sample MR results from all sensitivity analyses were directionally consistent with the main IVW estimate, though for several the CIs were very wide; IVW, MR-PRESSO and MR-TRYX supported an inverse association of maternal insomnia with offspring birthweight with CIs that did not include the null (Fig. 5). The MR-Egger intercept suggested little evidence of unbalanced horizontal pleiotropy (*p*-value = 0.732 for dataset A on B and 0.763 for B on A; full results in Supplementary Fig. 4). Whilst between SNP heterogeneity was less when MR-TRYX was used (in comparison to the IVW analyses) the point estimates were very similar between it and IVW (Fig. 5 and Supplementary Fig. 5). However, offspring genotypes (which were missing from UKB) could be a potential source of horizontal pleiotropy as we conducted MR between generations. Such bias cannot be mitigated by these pleiotropy robust methods but could be corrected by adjusting for paternal and offspring genotypes, where trios’ data are available [14].

## Discussion

As GWAS explore a larger range of phenotypes with larger sample sizes, the possibility that proposed genetic IVs for a specific exposure will be found to associate with many other traits at genome-wide significant threshold levels increases [6, 7]. This may make the selection of valid IVs for MR challenging. In this paper we have identified and described five scenarios that could result in associations of proposed genetic IVs with non-exposure traits. These are confounding, vertical pleiotropy, horizontal pleiotropy, reverse causality and selection. We provide a set of recommendations for exploring associations of the proposed genetic IV with non-exposure traits, identifying which scenario is most plausible, whether the MR result is likely to be biased and how any bias might be minimised. These recommendations are not fully covered in existing published MR guidelines, and supplement those [2, 3, 9]. We demonstrate the use of these recommendations in an applied example exploring the effect of maternal insomnia on birthweight.

In our example we found that our proposed IV for insomnia (a PRS of 80 SNPs) was associated with all six of our a priori selected risk factors for birthweight (height, BMI, age at first live birth, education, frequency of alcohol intake, ever smoking). Our results suggest that some of the association of the PRS with age at first live birth might be due to confounding by population structure. Consistent with previous MR studies [42, 45, 85, 86], our results suggested that associations of the PRS with height and BMI were less likely to reflect horizontal pleiotropy. Therefore, we concluded in this example associations of the insomnia PRS with height and BMI were unlikely to bias the main MR result. Age at first live birth, education and ever smoking were plausible sources of horizontal pleiotropy, and after adjusting for these in MVMR there was little evidence for a causal effect of maternal insomnia on offspring birthweight. Additional analyses in which we used a phenome-wide approach to identify associations of the 80 SNPs in the PRS with non-exposure traits, suggests we might have missed some key specific pleiotropic paths (e.g. related to mental, musculoskeletal, respiratory and general health). For these we relied on sensitivity analyses (see Table 2) to explore and control for any potential bias due to unbalanced horizontal pleiotropy, which largely suggested the little evidence of such bias. Depending on the particular research focus the reassurance from sensitivity analyses may be sufficient, and no further exploration of the specific role of non-exposure traits that the genetic IV is related to is required. However, we would suggest that any researchers exploring the effect of insomnia using MR might want to undertake multivariable regression analyses for some of the non-exposure traits influenced by insomnia SNPs that we have identified, particularly if sensitivity analyses for their outcomes suggest unbalanced horizontal pleiotropy.

Limitations in MR studies of prenatal exposures have been summarized in a recent review [87]. Our real data example was used for illustrative purposes rather than to make a statement about the effect of insomnia on birthweight, but we feel it is relevant to briefly acknowledge limitations specific to this example. We are assuming that the proposed genetic IVs for insomnia outside of pregnancy are valid for insomnia during pregnancy, which may not be the case if our SNPs–insomnia effects are influenced by the physiological change of pregnancy. MR estimates might be biased if the IVs we have proposed do not relation to insomnia in pregnancy as they do outside of pregnancy [17]. When exploring pregnancy exposures it is important, where possible to compare the proposed genetic IV—exposure association from the GWAS in non-pregnant participants with the equivalent association in pregnant women [14]. This has consistently been shown to be the case for blood pressure, lipids and glucose [42], but we cannot assume that it is the case for insomnia. Furthermore, studies of pregnancy exposures on offspring outcomes are often concerned with intrauterine exposure being a critical or sensitive period, and not influenced by preconceptual or postnatal exposure [14]. In this example if maternal insomnia (or genetic variants related to it) influenced oocytes, but not to the extent that they would make them inviable for fertilisation, and that effect on oocytes influence fetal growth our MR estimate would potentially reflect preconceptual as well as intrauterine effects. We are not aware of any evidence of maternal sleep quality influencing oocytes and as our outcome is birthweight, which could not be influenced by maternal postnatal insomnia. Our MR estimates might also be biased by overfitting data given the overlap participants between the GWAS used to select genetic IVs for insomnia and our MR analyses [58], and by the selective response in UKB [88], or self-report by women many years later of weight of their first live-born child. By definition our study only includes women who have delivered at least one live born baby. However, we are not aware of strong evidence of insomnia or our proposed genetic IVs for insomnia influencing infertility or number of live births [82, 89], and they were only weakly associated with a higher odds of having live born babies, indicating any bias due to only selecting pregnant women would be small [34, 36]. Additionally, we could not rule out the possibility that any of the additional non-exposure traits found in our phenome-wide scans resulted from confounding, horizontal pleiotropy or selection. Our MR estimates could also be influenced by violation of the fourth IV assumption [2]. In short, the real data example is an illustration of how to work through our recommendations and we are not suggesting that the effect estimate for this example is necessarily a valid causal effect of insomnia on birthweight.

To conclude we have highlighted an issue in MR that is likely to become increasingly common—that of finding multiple associations of proposed genetic IVs with non-exposure traits—and provide descriptions of the scenarios that could result in this, together with recommendations for how to explore whether such associations exist and what to do when they are found. These recommendations complement current MR guidelines [2, 3, 9]. Though not explored here, it is always helpful to triangulate MR results with other methods that have different key sources of bias to estimate causal effects [13]. Consistency of results across different methods increases confidence in the results, even in the presence of remaining concerns about genetic IV validity.

## Supplementary Information

Below is the link to the electronic supplementary material.

Supplementary file1 (DOCX 491 KB)

Supplementary file2 (XLSX 2080 KB)
