# Supplementary material for: Exploring and mitigating potential bias when genetic instrumental variables are associated with multiple non-exposure traits in Mendelian randomization
Source: Eur J Epidemiol. 2022 May 27;37(7):683–700. doi: 10.1007/s10654-022-00874-5 (PMC9329407; doi:10.1007/s10654-022-00874-5)
Supplement: Supplementary file 1 — Supplementary file1 (DOCX 491 KB) [file 10654_2022_874_MOESM1_ESM.docx]

**Exploring and mitigating potential bias when genetic instrumental variables are associated with multiple non-exposure traits in Mendelian randomization**

Qian Yang,^1,2^* Eleanor Sanderson,^1,2^ Kate Tilling,^1,2,3^ M Carolina Borges,^1,2^† Deborah A Lawlor^1,2,3^†

^1^MRC Integrative Epidemiology Unit at the University of Bristol, Bristol, UK

^2^Population Health Sciences, Bristol Medical School, University of Bristol, Bristol, UK

^3^National Institute for Health Research Bristol Biomedical Center, University Hospitals Bristol NHS Foundation Trust and University of Bristol, Bristol, UK

*Corresponding author

Email: qian.yang@bristol.ac.uk

†Deborah A Lawlor and M Carolina Borges made equal contributions and are joint senior authors.

**SUPPLEMENTARY MATERIAL**

Supplementary Methods

Supplementary Fig. 1 A directed acyclic graph for the effect of maternal insomnia on offspring birthweight

Supplementary Fig. 2 The number of participants in one- and two-sample Mendelian randomization of our real data example

Supplementary Fig. 3 Distributions of polygenetic risk score for insomnia across 22 UK Biobank assessment centres

Supplementary Fig. 4 Scatter plots of two-sample Mendelian randomization for the effect of insomnia on birthweight using MR-Base

Supplementary Fig. 5 Scatter plots of two-sample Mendelian randomization for the effect of insomnia on birthweight using MR-TRYX

Supplementary Table 1. The UK Biobank data fields of variables used in this study

Supplementary Table 2. Results for age at first live birth adjusting for genetic array, participants’ age and birthplace, and UK Biobank assessment centres

Supplementary Table 3. Strengths of polygenetic risk score (PRS)

Supplementary Data 1. Information of genetic variants used as instrumental variables in real data example

Supplementary Data 2. Genome-wide significant associations of insomnia-related variants extracted from Phenoscanner

Supplementary Data 3. Original output from ‘some invalid some Valid IV Estimator’ for the effect of insomnia on birthweight

*Supplementary Data are in a separate file.

**Supplementary Methods**

***Genetic instrumental variables (IVs) for insomnia***

We used single nucleotide polymorphisms (SNPs) identified by the largest genome-wide association study (GWAS) combining UK Biobank (UKB) with 23andMe participants [1] due to its sample size (N=709 986 women), provision of female-specific results and a low proportion (29%) of overlapped participants with UKB women to try to avoid winner’s curse [2]. This GWAS reported 83 female-specific SNPs robustly associated with insomnia (P-value < 5×10^-8^). From these, we removed 3 SNPs that were correlated to other variants using MR-Base ‘clumping’ function (R^2^=0.01, referring to the European samples from the 1000 genomes project) [3], and then extracted genotypes from UKB for the remaining 80 SNPs. Given no external (to UKB) weights were available and the 80 SNPs had similar effect sizes in the GWAS [1], we derived an unweighted polygenic risk score (PRS), by adding up the number of insomnia risk-raising alleles [4]. We used the PRS as the IV in one-sample Mendelian randomization (MR).

***Genetic IVs for height, body mass index (BMI), age at first live birth, education, frequency of alcohol intake and ever smoking***

We aimed to use GWAS conducted in only women of European descent which did not overlap with UKB. We searched for genome-wide significant (P-value < 5×10^-8^) SNPs and performed ‘clumping’ (R^2^=0.01, referring to the European samples from the 1000 genomes project) for those traits on MR-Base, if their GWAS were included. The Genetic Investigation of Anthropometric Traits consortium identified 54 and 38 SNPs associated with female height (N=147 746, dataset ID ‘97’ on MR-Base) [5] and female BMI (N=171 977, dataset ID ‘974’ on MR-Base) [6], respectively. Social Science Genetic Association Consortium identified 6 and 19 SNPs associated with female age at first live birth (N=154 839, not on MR-Base) [7] and female year of schooling (N=182,286, dataset ID ‘1011’ on MR-Base) [8], respectively. These four GWAS were conducted in non-UKB settings. Neale Lab’s GWAS identified 44 and 40 SNPs associated with frequency of alcohol intake (N=336 965, dataset ID ‘UKB-a:25’ on MR-Base) and ever smoking (N=336 067, dataset ID ‘UKB-a:236’ on MR-Base) in UKB men and women [9]. We derived weighted PRS as IVs for one-sample MR using effect sizes reported by the corresponding GWAS as the weights. To minimize bias from internal weight [10], we also followed previous MR studies [11, 12] to repeat our analyses using rs1229984 (*ADH1B*) and rs698 (*ADH1C*) for frequency of alcohol intake and rs6265 (*BDNF*) for ever smoking, which were identified in non-UKB settings [13, 14].

***Exploring the role of population stratification***

Individual level data allow us to check for population stratification. We tested associations of insomnia PRS with maternal age at recruitment and birthplace (including longitude and latitude) and compared means of PRS across 22 UKB study centres using ANOVA. We also compared each non-exposure trait association according to PRS using (i) a crude model, (ii) a model adjusting for genetic array (as UK Biobank participants were genotyped on either of two arrays) and top 40 principal components, (iii) a model further adjusting for participants’ age at recruitment and birthplace, and (iv) a model adjusting for all these covariates plus UKB study centres. We obtained differences in mean non-exposure traits per allele increase in PRS from a linear regression, except for ever smoking where we applied logistic regression.

We do not have a specific variable for “ever became pregnant”. Therefore, to explore potential bias due to selection on pregnancy, we examined the association of insomnia PRS with having live born babies using logistic regressions, and the association of having insomnia with having live born babies using two-stage least squares, adjusting for genetic array, top 40 principal components, participants’ age at recruitment and birthplace, and UKB study centres.

***Univariable MR to assess bias due to horizontal pleiotropy***

We used univariable MR to explore (i) the associations of the six non-exposure traits (W) with birthweight (Y), (ii) the causal directions between insomnia (X) and W, and (3) the association of X with Y for comparison. In one-sample setting, we applied two-stage least squares (TSLS) [15]. In the first stage, the exposure (a continuous or binary variable, $x_{i}$) was regressed on its PRS ($z_{i}$) in a linear model (equation 1). In the second stage, the outcome (a continuous or binary, $y_{i}$) was regressed on the fitted values for exposure ($\hat{x_{i}}$) from the first stage in a linear model (equation 2). $\varepsilon_{x_{i}}$ and $\varepsilon_{y_{i}}$ represented the independent error terms. We used a linear model rather than logit model (which would be more common with a binary exposure) to avoid non-collapsibility of odds ratio and difficult interpretation of the units (e.g. per doubling of probability of binary exposure) [16].

$$x_{i}= \alpha_{0}+ \alpha_{1}\times z_{i}+ \varepsilon_{x_{i}} (equation 1)$$

$$y_{i}= \beta_{0}+ \beta_{1}\times\hat{x_{i}}+ \varepsilon_{y_{i}} (equation 2)$$

The unit of TSLS estimates correspond to difference in mean per one unit increase in the exposure. Specifically, the unit of birthweight is gram. We standardized height, BMI and age at first live birth to make their estimates comparable to each other, and 1 SD is equivalent to 6 cm in height, 5 kg/m^2^ in BMI and 5 years in age at first live birth. For education and frequency of alcohol intake, which are ordered categorical, one-unit increase represents one level change from a lower category to a higher category. For binary variables, one-unit increase in ever smoking represents comparing ever smokers to never smokers, and one-unit increase in insomnia represents comparing participants reporting that they “usually” experience insomnia to “sometimes/rarely/never”. We obtained differences in mean (or absolute differences in risk of binary variables) together with 95% confidence intervals using ‘ívreg’ package in R, which can give a correct standard error considering the uncertainty in both regression stages.

We examined the strength of PRS via F-statistic in each first stage regression [17], with results shown in Supplementary Table 3. For the effect of insomnia on birthweight, we assessed between SNP heterogeneity via Sargan test which is an ‘overidentifying’ test and need individual SNPs to be IVs in TSLS. Rejection of its null hypothesis suggests at least one invalid IV [18].

Since the GWAS of insomnia included UKB [1], we followed a previous study that had the same problem[19] to conduct our two-sample MR. We randomly split our UKB women (N=208 171) into two sets (N_A_=104 041 and N_B_=104 130, see Supplementary Fig. 3). In each dataset, we obtained SNP-specific associations with each trait (i.e. IV_X_-X, IV_X_-Y, IV_X_-W, IV_W_-X, IV_W_-Y and IV_W_-W) by running linear regressions. For W-Y associations, we used IV_W_-W from dataset A and IV_W_-Y from dataset B (A on B) and vice versa (B on A) in inverse variance weighted (IVW) analyses. Similarly, for W-X associations, we used IV_W_-W from dataset A and IV_W_-X from dataset B (A on B) and vice versa (B on A) in IVW; for X-W associations, we used IV_X_-X from dataset A and IV_X_-W from dataset B (A on B) and vice versa (B on A) in IVW; for the X-Y association, we used IV_X_-X from dataset A and IV_X_-Y from dataset B (A on B) and vice versa (B on A) in IVW. We used ‘TwoSampleMR’ package in R to conduct the IVW analyses. At last, we pooled the MR estimates from the two together for each association. For the effect of X on Y, we assessed between SNP heterogeneity via Cochran’s Q statistic via MR-Base.

In one- and two-sample MR, we included genetic array and top 40 principal components as covariates, given further adjustments for participants’ age, birthplace and study centre showed relatively similar associations in Fig. 1. We conducted sensitivity analyses for age at first live birth to include those as covariates, with results shown in Supplementary Table 2.

***Multivariable MR to account for horizontal pleiotropy***

We would conduct multivariable MR of effects of (i) insomnia and age at first live birth, (ii) insomnia and education, (iii) insomnia and ever smoking, and (iv) insomnia, age at first live birth, education and ever smoking on birthweight. In one-sample setting, we used PRS for X and W as their IVs in TSLS, and their strengths were assessed via F-statistics (results shown in Supplementary Table 2) [20]. In two-sample setting, we conducted multivariable MR using IV_X_-X, IV_X_-W, IV_W_-W, IV_W_-X from dataset A and IV_X_-Y, IV_W_-Y from dataset B (A on B) and vice versa (B on A). Finally, we pooled the MR estimates from the two together for each insomnia-birthweight association.

***Sensitivity analyses***

In one-sample MR, we applied some invalid some valid instrumental variable estimator (sisVIVE) by using the ‘sisVIVE’ package in R.[21] The package requires our data in a matrix format with 3 columns (i.e. PRS, insomnia, birthweight) × 165 184 rows (i.e. the number of participants). sisVIVE can identify one invalid IV at a time and provide a difference in mean birthweight with correction for that. Since we have 80 variants for insomnia, sisVIVE provided 80 differences in mean birthweight with correction for 0, 1, 2 …… 78 and 79 invalid IVs (full results in Supplementary Data 2). We also used ‘genius’ package in R to obtain estimates from MR G-Estimation under No Interaction with Unmeasured Selection (MR-GENIUS) [22], which requires PRS, insomnia and birthweight data at the individual level.

In two-sample MR, we used ‘TwoSampleMR’ package in R [3] to obtain estimates from MR-Egger [23], weighted median [24] and weighted mode [25] approaches, ‘MRPRESSO’ package in R to obtain estimates from MR Pleiotropy RESidual Sum and Outlier method [26], and ‘tryx’ package in R to obtain estimates from MR Treasure Your eXceptions [27]. In each method, we used IV_X_-X from dataset A and IV_X_-Y from dataset B (A on B) and vice versa (B on A), and finally meta-analysed the MR estimates from the two.

**Supplementary Fig. 1 A directed acyclic graph for the effect of maternal insomnia on offspring birthweight**

**
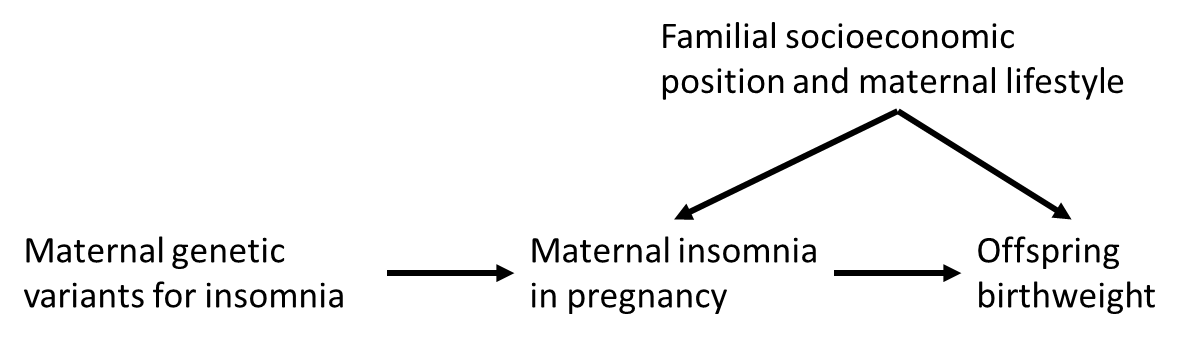
**

The three key assumptions are:

a. Maternal genetic variants for insomnia are robustly related to maternal insomnia in pregnancy

b. Confounders of the maternal insomnia in pregnancy – offspring birthweight association (e.g. familial socioeconomic position and maternal lifestyle) are not associated with maternal genetic variants for insomnia.

c. Maternal genetic variants for insomnia are not associated with offspring birthweight other than potentially through its association with maternal insomnia in pregnancy.

**Supplementary Fig. 2 The number of participants in one- and two-sample Mendelian randomization of our real data example**

**
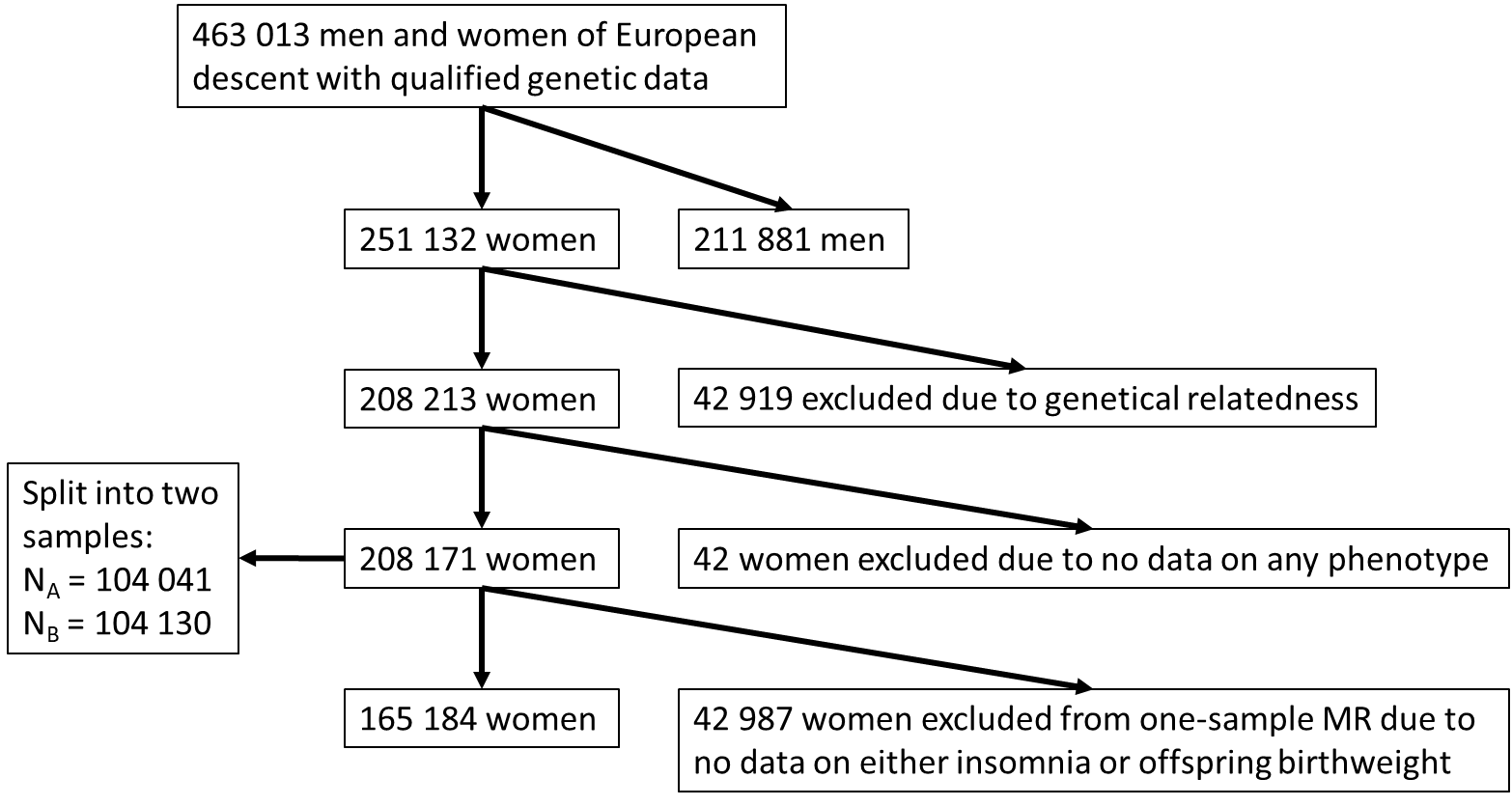
**

**Supplementary Fig. 3 Distributions of polygenetic risk score for insomnia across 22 UK Biobank assessment centres**

**
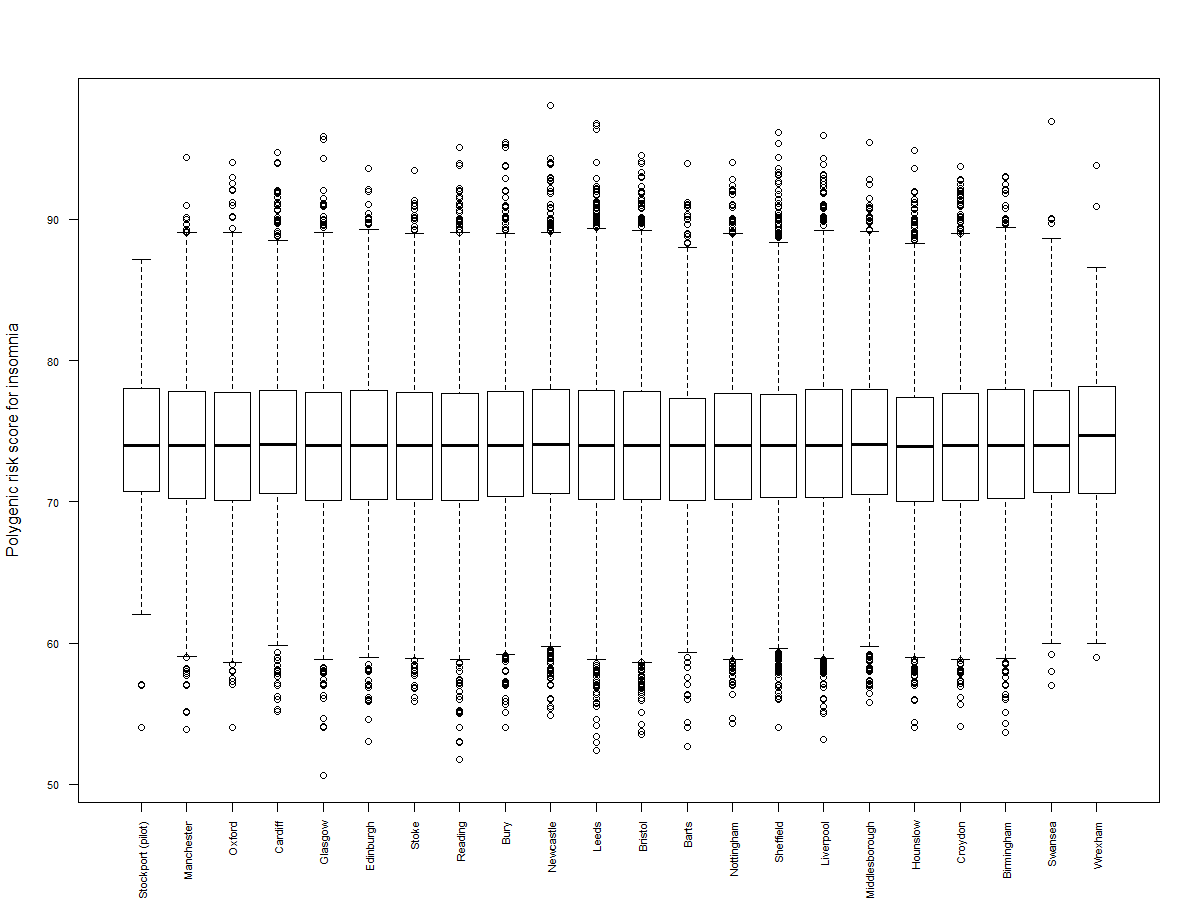
**

Boxplot shows median, interquartile range (IQR), minimum (25^th^ percentile – 1.5*IQR), maximum (75^th^ percentile + 1.5*IQR) and outliers.

**Supplementary Fig. 4 Scatter plots of two-sample Mendelian randomization for the effect of insomnia on birthweight using MR-Base**

(a) Dataset A on dataset B (Inverse variance weighted Q statistic = 101, P = 0.048; MR-Egger intercept = 0.275, SE = 0.797, P = 0.732)


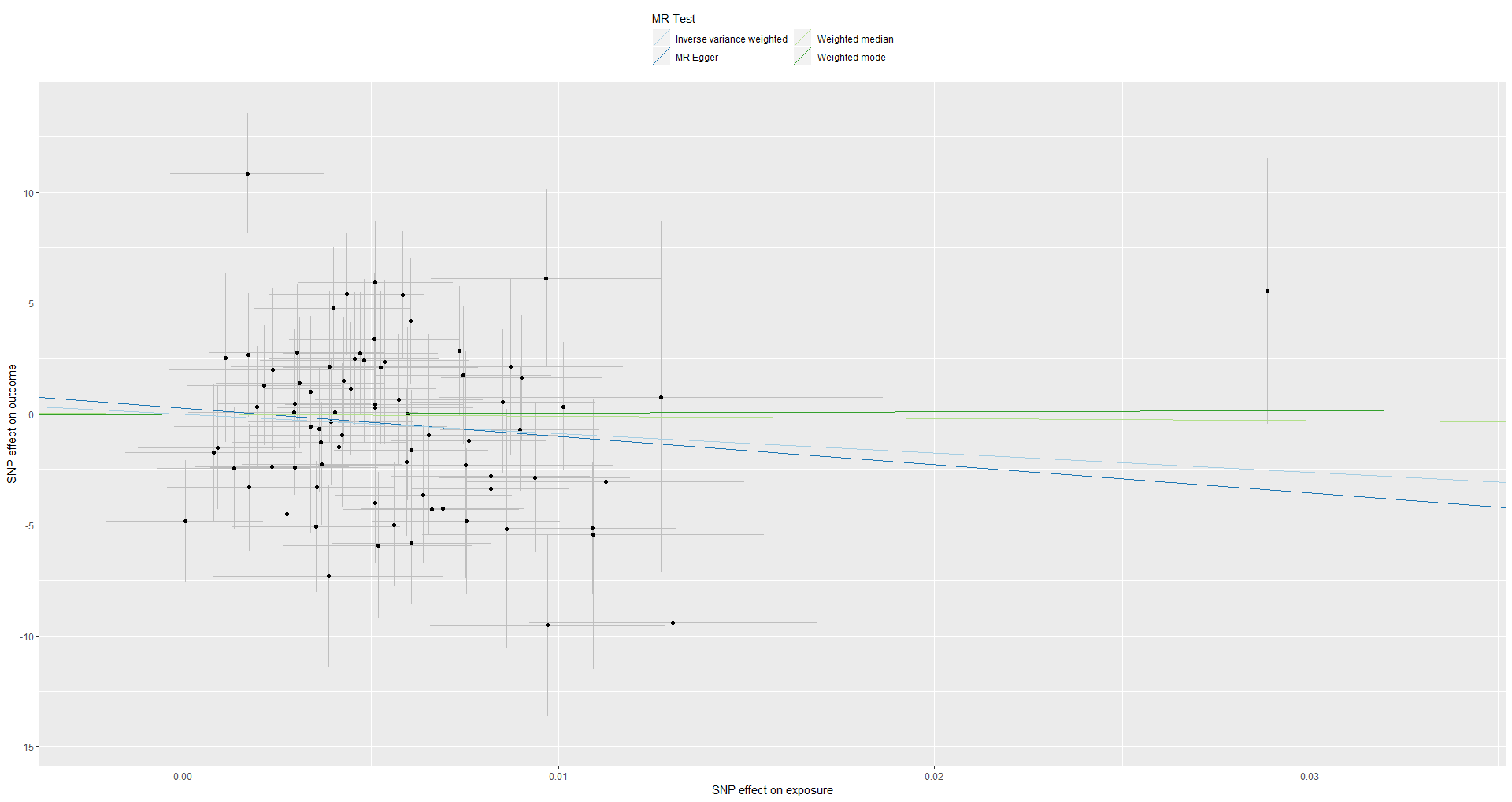


(b) Dataset B on dataset A (Inverse variance weighted Q statistic = 157, P = 3.85×10^-7^; MR-Egger intercept = -0.315, SE = 1.041, P = 0.763)


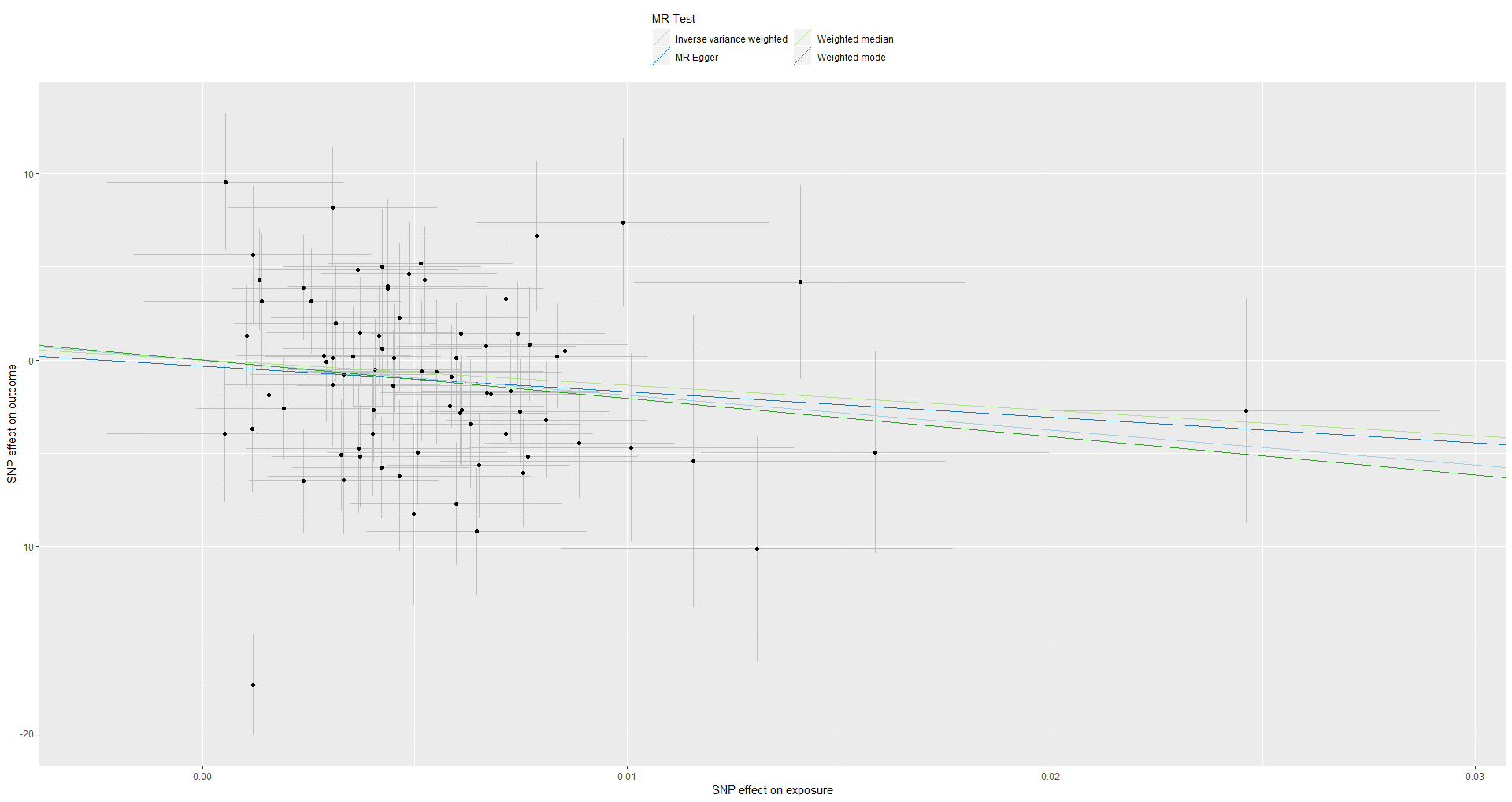


**Supplementary Fig. 5 Scatter plots of two-sample Mendelian randomization for the effect of insomnia on birthweight using MR-TRYX**

(a) Dataset A on dataset B (Q statistic = 84 in the two outliers removed models and Q = 91 in the outlier adjusted model)


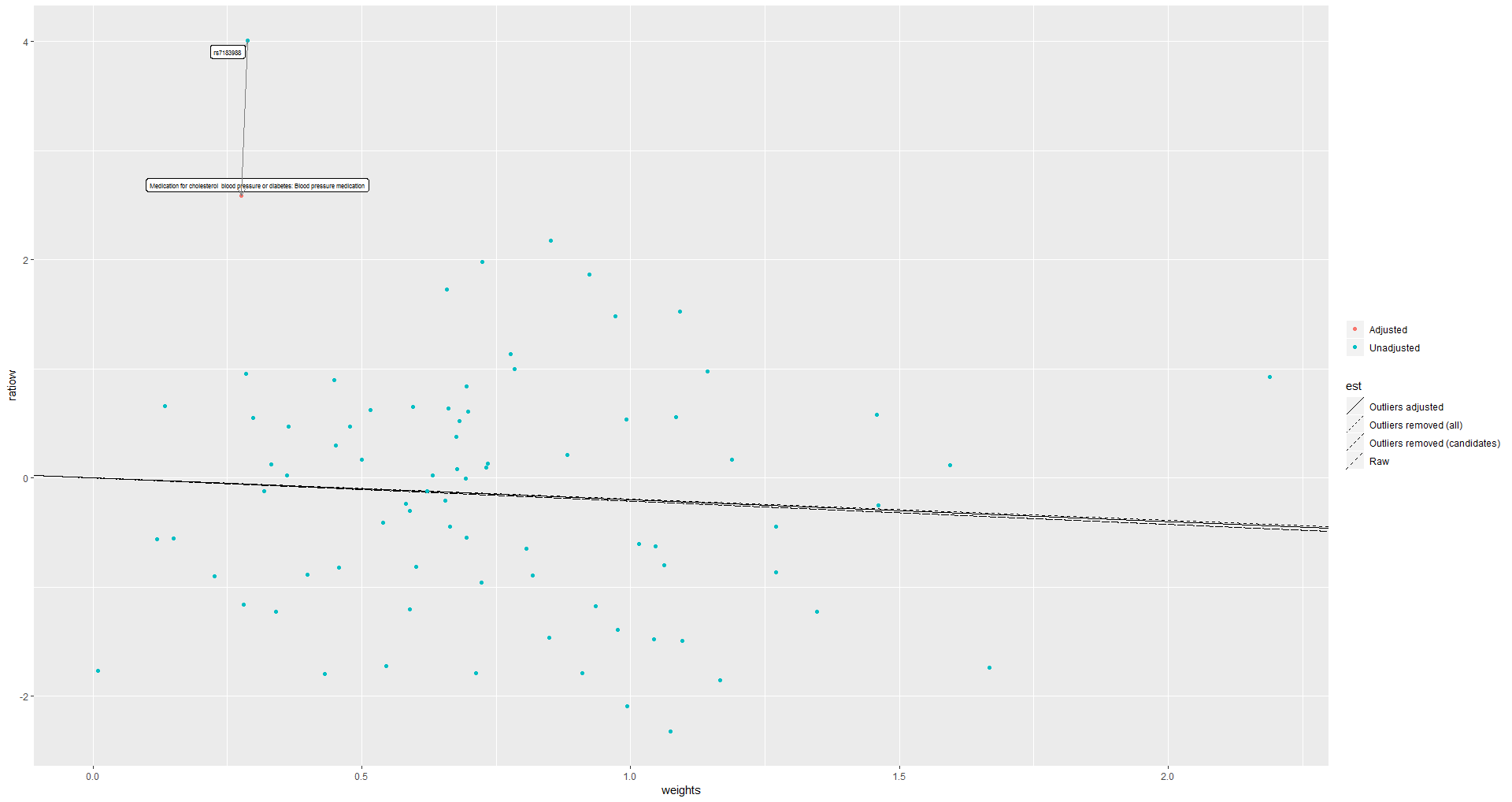


(b) Dataset B on dataset A (Q = 117 in the two outliers removed models and Q = 141 in the outlier adjusted model)


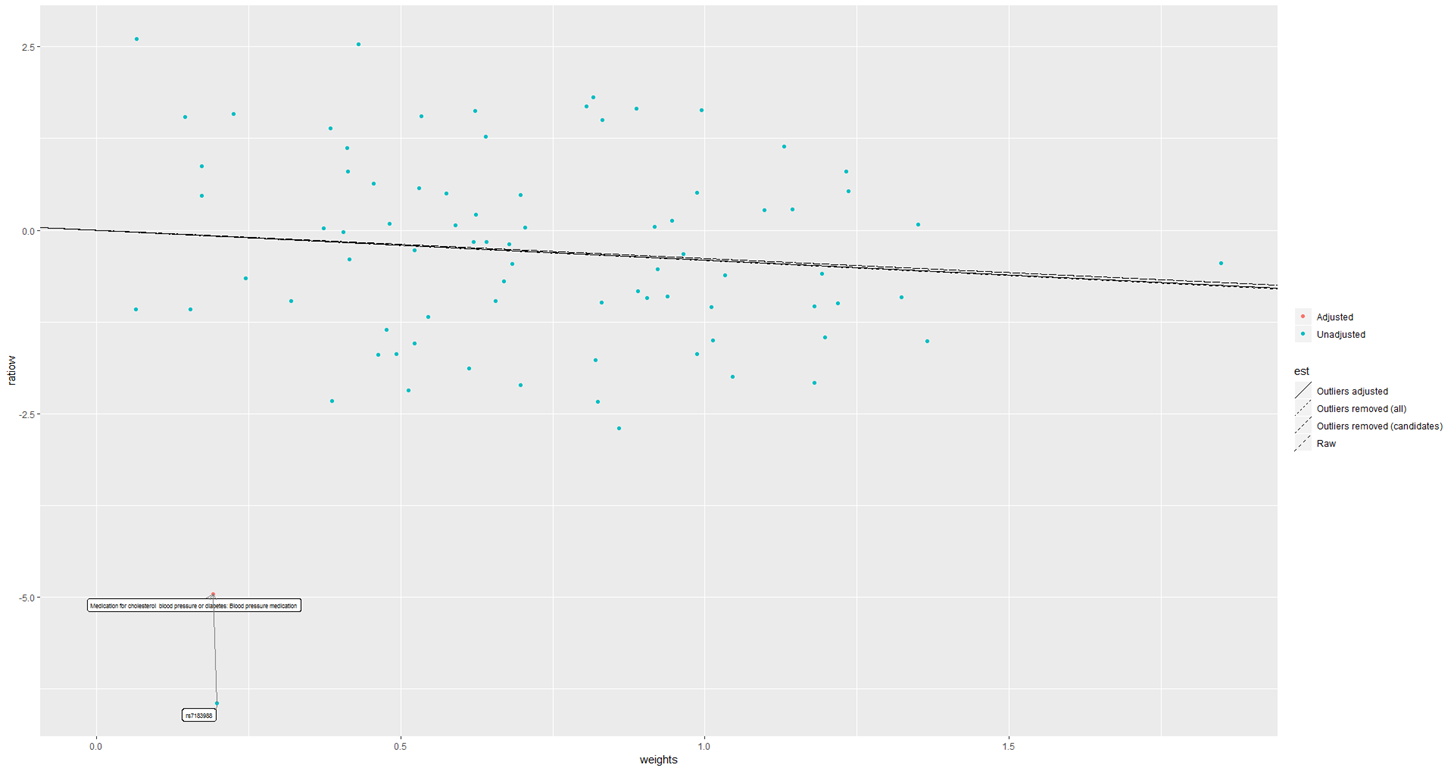


**Supplementary Table 1. The UK Biobank data fields of variables used in this study**

| **Variable** | **Field ID** | **Code in this study** | **N** |
| --- | --- | --- | --- |
| ***Exposure*** |  |  |  |
| Maternal insomnia | 1200 | “Do you have trouble falling asleep at night or do you wake up in the middle of the night?”, with answers “never/rarely”=0, “sometimes”=0 and “usually”=1. | 207 924 |
| ***Outcome*** |  |  |  |
| Offspring birthweight | 2744 | “What was the birth weight of your first child in pounds? (do not include twins)”, with answers ranging from 2 to 15. We converted its unit to grams. | 165 254 |
| ***Maternal characteristics*** | | | |
| Height | 50 | A continuous variable, mean≈163 cm, SD≈6 cm | 207 704 |
| Body mass index | 21001 | A continuous variable, mean≈27 kg/m^2^, SD≈5 kg/m^2^ | 207 477 |
| Age at first live birth | 2754 | “How old were you when you had your FIRST child?” A continuous variable, mean≈25 years, SD≈5 years | 140 503 |
| Education | 6138 | “Which of the following qualifications do you have? (You can select more than one)”, with answers “College or University degree”=3, “A levels/AS levels or equivalent”=2, “O levels/GCSEs or equivalent”=1, “CSEs or equivalent”=1, “NVQ or HND or HNC or equivalent”=1, “Other professional qualifications eg: nursing, teaching”=2, “None of the above”=1. | 206 055 |
| Frequency of alcohol intake | 1558 | “About how often do you drink alcohol?”, with answers “Daily or almost daily”=6, “Three or four times a week”=5, “Once or twice a week”=4, “One to three times a month”=3, “Special occasions only”=2, “Never”=1. | 207 872 |
| Ever smoking | 20116 | “Never” = 0, “Previous” = 1, “Current” = 1. | 207 281 |
| ***Covariates*** |  |  |  |
| Assessment centre | 54 | A categorical variable | 208 171 |
| Age | 21003 | A continuous variable | 208 171 |
| Place of birth in UK | 129, 130 | Two continuous variables (longitude and latitude) | 189 470 |
| ***Test for selection bias*** |  |  |  |
| Having live born babies | 2734 | “How many children have you given birth to? (Please include live births only)”, with answers “0”=0, “1” or more=1, “Prefer not to answer”=NA | 207,885 |

We coded “Prefer not to answer” as missing. All phenotypic values in the analyses were no less than 0. Details of how these variables were assessed can be found on <http://biobank.ctsu.ox.ac.uk/crystal/search.cgi>.

**Supplementary Table 2. Results for age at first live birth adjusting for genetic array, participants’ age and birthplace, and UK Biobank assessment centres**

| **Mendelian randomization** | **MR design** | **Model 1** | **Model 2** |
| --- | --- | --- | --- |
| ***Univariable*** |  |  |  |
| Age at first live birth (SD)→ birthweight (grams) | One-sample | 85.2 (-11.0, 181.5) | 96.1 (-5.1, 197.3) |
|  | Two-sample | 87.4 (-9.5, 184.4) | 91.2 (-7.7, 190.0) |
| Age at first live birth (SD)→ insomnia | One-sample | -0.084 (-0.166, -0.002) | -0.077 (-0.163, 0.009) |
|  | Two-sample | -0.123 (-0.197, -0.056) | -0.148 (-0.222, -0.073) |
| Insomnia→ age at first live birth (SD) | One-sample | -1.025 (-1.213, -0.838) | -0.991 (-1.178, -0.804) |
|  | Two-sample | -0.826 (-1.048, -0.605) | -0.769 (-0.982, -0.555) |
| ***Multivariable*** |  |  |  |
| Insomnia + age at first live birth→ birthweight (grams) | One-sample | 18.0 (-131.3, 167.2) | 23.1 (-127.6, 173.8) |
|  | Two-sample | -111.9 (-255.7, 32.0) | -99.4 (-244.1, 45.4) |
| Insomnia + age at first live birth + education + being ever smokers→ birthweight (grams) | One-sample | -37.5 (-224.4, 149.4) | -56.5 (-243.0, 130.0) |
|  | Two-sample | -52.0 (-155.2, 51.2) | -48.7 (-152.9, 55.4) |

Estimates are differences in mean outcome per unit increase in exposure. One unit of age at first live birth is 1 SD (5 years) in univariable Mendelian randomization and 1 year in multivariable Mendelian randomization. Education has 3 levels; being ever smokers and insomnia are binary. Model 1 adjusted for genetic array and top 40 principal components (presented in Figure 2), while Model 2 further adjusted for participants’ age, birthplace and assessment centre.

**Supplementary Table 3. Strengths of polygenetic risk score (PRS)**

| **Mendelian randomization** | **F-statistics ^a^** | |
| --- | --- | --- |
| ***Univariable*** |  |  |
| PRS of 54 variants→ height→ birthweight | 9376 | |
| PRS of 38 variants→ body mass index→ birthweight | 1946 | |
| PRS of 6 variants→ age at first live birth→ birthweight | 147 | |
| PRS of 19 variants→ education→ birthweight | 574 | |
| PRS of 44 variants→ alcohol consumption frequency→ birthweight | 1264 | |
| PRS of 2 variants→ alcohol consumption frequency→ birthweight | 181 ^b^ | |
| PRS of 40 variants→ being ever smokers→ birthweight | 604 | |
| rs6265 (*BDNF*)→ being ever smokers→ birthweight | 8 ^c^ | |
| PRS of 54 variants→ height→ insomnia | 11 886 | |
| PRS of 38 variants→ body mass index→ insomnia | 2668 | |
| PRS of 6 variants→ age at first live birth→ insomnia | 149 | |
| PRS of 19 variants→ education→ insomnia | 744 | |
| PRS of 44 variants→ alcohol consumption frequency→ insomnia | 1590 | |
| PRS of 2 variants→ alcohol consumption frequency→ insomnia | 212 ^b^ | |
| PRS of 40 variants→ being ever smokers→ insomnia | 777 | |
| rs6265 (*BDNF*)→ being ever smokers→ insomnia | 7 ^c^ | |
| PRS of 80 variants→ insomnia→ height | 686 | |
| PRS of 80 variants→ insomnia→ body mass index | 682 | |
| PRS of 80 variants→ insomnia→ age at first live birth | 494 | |
| PRS of 80 variants→ insomnia→ education | 671 | |
| PRS of 80 variants→ insomnia→ alcohol consumption frequency | 690 | |
| PRS of 80 variants→ insomnia→ being ever smokers | 688 | |
| PRS of 80 variants ^d^ → insomnia→ birthweight | 591 | |
| ***Multivariable*** | ***Unconditional*** | ***Conditional*** |
| PRS_1_ + PRS_2_→ insomnia + age at first live birth→ birthweight |  |  |
| PRS_1_ of 80 variants for insomnia | 245 | 73 |
| PRS_2_ of 6 variants for age at first live birth | 132 | 64 |
| PRS_1_ + PRS_3_→ insomnia + education→ birthweight |  |  |
| PRS_1_ of 80 variants for insomnia | 289 | 286 |
| PRS_3_ of 19 variants for education | 299 | 368 |
| PRS_1_ + PRS_4_→ insomnia + being ever smokers→ birthweight |  |  |
| PRS_1_ of 80 variants for insomnia | 300 | 299 |
| PRS_4_ of 40 variants for being ever smokers | 320 | 333 |
| PRS_1_ + PRS_2_ + PRS_3_ + PRS_4_→ insomnia + age at first live birth + education + being ever smokers→ birthweight |  |  |
| PRS_1_ of 80 variants for insomnia | 123 | 7 |
| PRS_2_ of 6 variants for age at first live birth | 105 | 5 |
| PRS_3_ of 19 variants for education | 150 | 5 |
| PRS_4_ of 40 variants for being ever smokers | 127 | 25 |

^a^ F-statistics for the same PRS may be slightly different due to different sample sizes in the analyses.

^b^ Little evidence was found for a causal effect.

^c^ No further analyses were conducted using this instrumental variable due to its small F-statistic.

^d^ When 80 individual SNPs were used as instrumental variables in two-stage least squares, Sargan test suggests invalid instrumental variables (P = 3×10^-8^).

**References**

1. Jansen PR, Watanabe K, Stringer S, et al. Genome-wide analysis of insomnia in 1,331,010 individuals identifies new risk loci and functional pathways. Nat Genet. 2019;51(3):394-403.

2. Lawlor DA. Commentary: Two-sample Mendelian randomization: opportunities and challenges. Int J Epidemiol. 2016;45(3):908-15.

3. Hemani G, Zheng J, Elsworth B, et al. The MR-Base platform supports systematic causal inference across the human phenome. Elife. 2018;7.

4. Burgess S, Thompson SG. Use of allele scores as instrumental variables for Mendelian randomization. Int J Epidemiol. 2013;42(4):1134-44.

5. Randall JC, Winkler TW, Kutalik Z, et al. Sex-stratified genome-wide association studies including 270,000 individuals show sexual dimorphism in genetic loci for anthropometric traits. PLoS Genet. 2013;9(6):e1003500.

6. Locke AE, Kahali B, Berndt SI, et al. Genetic studies of body mass index yield new insights for obesity biology. Nature. 2015;518(7538):197-206.

7. Barban N, Jansen R, de Vlaming R, et al. Genome-wide analysis identifies 12 loci influencing human reproductive behavior. Nat Genet. 2016;48(12):1462-72.

8. Okbay A, Beauchamp JP, Fontana MA, et al. Genome-wide association study identifies 74 loci associated with educational attainment. Nature. 2016;533(7604):539-42.

9. We're thrilled to announce an updated GWAS analysis of the UK Biobank. The Neale Lab. 2018. <http://www.nealelab.is/uk-biobank>. Accessed 17 Oct 2019.

10. Hartwig FP, Davies NM. Why internal weights should be avoided (not only) in MR-Egger regression. Int J Epidemiol. 2016;45(5):1676-8.

11. Lawlor DA, Nordestgaard BG, Benn M, Zuccolo L, Tybjaerg-Hansen A, Davey Smith G. Exploring causal associations between alcohol and coronary heart disease risk factors: findings from a Mendelian randomization study in the Copenhagen General Population Study. Eur Heart J. 2013;34(32):2519-28.

12. Gibson M, Munafo MR, Taylor AE, Treur JL. Evidence for Genetic Correlations and Bidirectional, Causal Effects Between Smoking and Sleep Behaviors. Nicotine Tob Res. 2019;21(6):731-8.

13. Tolstrup JS, Nordestgaard BG, Rasmussen S, Tybjaerg-Hansen A, Gronbaek M. Alcoholism and alcohol drinking habits predicted from alcohol dehydrogenase genes. Pharmacogenomics J. 2008;8(3):220-7.

14. Genome-wide meta-analyses identify multiple loci associated with smoking behavior. Nat Genet. 2010;42(5):441-7.

15. Burgess S, Dudbridge F, Thompson SG. Combining information on multiple instrumental variables in Mendelian randomization: comparison of allele score and summarized data methods. Stat Med. 2016;35(11):1880-906.

16. Burgess S, Labrecque JA. Mendelian randomization with a binary exposure variable: interpretation and presentation of causal estimates. Eur J Epidemiol. 2018;33(10):947-52.

17. Burgess S, Small DS, Thompson SG. A review of instrumental variable estimators for Mendelian randomization. Stat Methods Med Res. 2017;26(5):2333-55.

18. Burgess S, Scott RA, Timpson NJ, Davey Smith G, Thompson SG. Using published data in Mendelian randomization: a blueprint for efficient identification of causal risk factors. Eur J Epidemiol. 2015;30(7):543-52.

19. Henry A, Katsoulis M, Masi S, et al. The relationship between sleep duration, cognition and dementia: a Mendelian randomization study. Int J Epidemiol. 2019;48(3):849-60.

20. Sanderson E, Davey Smith G, Windmeijer F, Bowden J. An examination of multivariable Mendelian randomization in the single-sample and two-sample summary data settings. Int J Epidemiol. 2018;48(3):713-27.

21. Kang H, Zhang A, Cai TT, Small DS. Instrumental Variables Estimation With Some Invalid Instruments and its Application to Mendelian Randomization. Journal of the American Statistical Association. 2016;111(513):132-44.

22. Tchetgen Tchetgen EJ, Sun B, Walter S. The GENIUS Approach to Robust Mendelian Randomization Inference. arXiv. 2019; <https://arxiv.org/abs/1709.07779>.

23. Bowden J, Davey Smith G, Burgess S. Mendelian randomization with invalid instruments: effect estimation and bias detection through Egger regression. Int J Epidemiol. 2015;44(2):512-25.

24. Bowden J, Davey Smith G, Haycock PC, Burgess S. Consistent Estimation in Mendelian Randomization with Some Invalid Instruments Using a Weighted Median Estimator. Genet Epidemiol. 2016;40(4):304-14.

25. Hartwig FP, Davey Smith G, Bowden J. Robust inference in summary data Mendelian randomization via the zero modal pleiotropy assumption. Int J Epidemiol. 2017;46(6):1985-98.

26. Verbanck M, Chen CY, Neale B, Do R. Detection of widespread horizontal pleiotropy in causal relationships inferred from Mendelian randomization between complex traits and diseases. Nat Genet. 2018;50(5):693-8.

27. Cho Y, Haycock PC, Sanderson E, et al. Exploiting horizontal pleiotropy to search for causal pathways within a Mendelian randomization framework. Nat Commun. 2020;11(1):1010.
